# Origin-dependent chemical profiles and quality differentiation of candied *Prunus mume*: Insights from metabolomics, GC–MS volatile profiling, and interpretable machine learning

**DOI:** 10.1016/j.fochx.2026.104391

**Published:** 2026-09-01

**Authors:** Jia-li Liao, Yi-xiao Xiao, Jia-xin Zhong, Yi-jing He, Wen-zhang Qian, Ji-cheng Chen, Jin-lin Guo, Shun Gao

**Affiliations:** a Department of Forestry, Faculty of Forestry, Sichuan Agricultural University, Chengdu 611130, China; b College of Food Science, Fujian Agriculture and Forest University, Fuzhou 350002, China; c Key Laboratory of Characteristic Chinese Medicine Resources in Southwest China, College of Pharmacy, Chengdu University of Traditional Chinese Medicine, Chengdu 611137, China; d Forest Ecology and Conservation in the Upper Reaches of the Yangtze River Key Laboratory of Sichuan Province & Sichuan Mt. Emei Forest Ecosystem National Observation and Research Station, Sichuan Agricultural University, Chengdu 611130, China

**Keywords:** Candied Prunus mume, Volatile organic compounds, Metabolomics, Quality differentiation, Geographical origin

## Abstract

*Prunus mume* is widely processed into candied products, but the food-chemical basis of origin-related quality variation remains unclear. Fruits from fourteen Chinese origins were processed into candied *P. mume* (CPM) under a unified candying regime and analyzed using quality-trait assays, gas chromatography–mass spectrometry (GC–MS) profiling of volatile organic compounds (VOCs), untargeted metabolomics, relative odor activity value (ROAV) analysis, weighted correlation network analysis (WCNA), and random forest–Shapley additive explanations (RF–SHAP). CPM showed origin-dependent differences in sugar–acid traits, antioxidant capacity, VOC profiles, and non-volatile metabolite profiles. ROAV and WCNA revealed shared aroma-active contributors and origin-associated volatile modules. The non-volatile metabolite profile showed abundance differences within a shared metabolite backbone, involving flavonoid glycosides, phenolic derivatives, carbohydrates/glycosides and organic-acid-related compounds. RF–SHAP prioritized differentiating metabolites, while cross-layer integration linked antioxidant-related variation to phenolic/flavonoid metabolism and sugar–acid variation to carbohydrates and organic acids. These findings provide a chemical basis for origin-oriented quality evaluation of CPM.

## Graphical abstract

**Figure:**
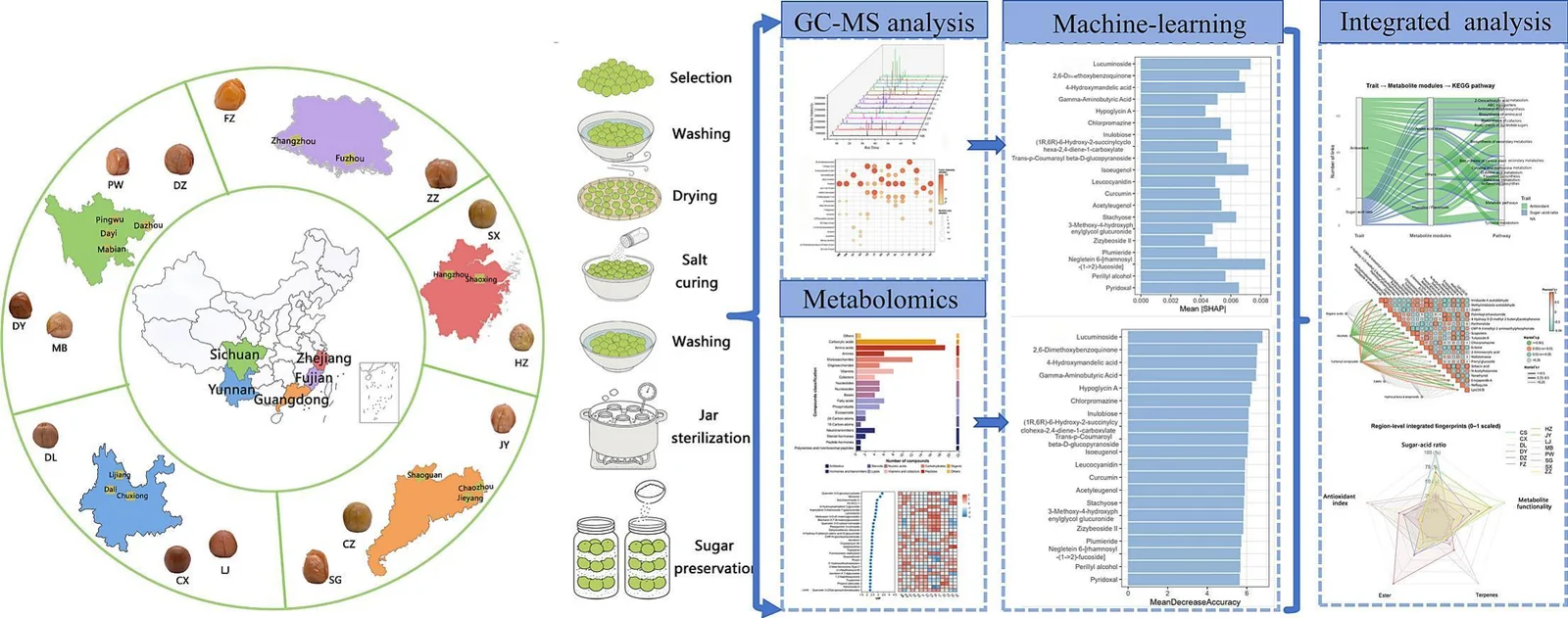

## 1 Introduction

Fruits are universally acknowledged as protective foods due to their rich vitamin content, playing an indispensable role in human nutrition. However, their inherently high moisture content renders them highly perishable, making them susceptible to spoilage at every stage of the post-harvest continuum, from harvesting to consumption (Sethi et al., 2025). China produces a large proportion of the world's fruit, yet post-harvest losses remain substantial. Studies estimate storage losses at 10–15% and transport-related losses at 5–10%, emphasizing the critical need for effective preservation methods to reduce wastage and maintain fruit quality (Cheng et al., 2024). Beyond minimizing post-harvest losses, fruit processing enables value addition and waste utilization. It plays a key role in supporting income and employment in agrarian economies, while processed fruit products possess strong potential for both domestic and international markets (Boateng, 2024; Wu et al., 2024). Therefore, fruit processing is not only a preservation strategy, but also a way to convert fresh fruit into value-added products with modified composition, taste, aroma, and shelf-life properties.

Candied fruits represent an important category of value-added fruit products, appreciated for their characteristic sweet-sour taste, distinctive aroma, desirable texture, and extended shelf life. The preparation of candied fruits involves impregnating whole fruits or fruit portions (such as peels and slices) with sugar, jaggery, or honey, often combined with partial drying or heating. This traditional preservation method relies on the high osmotic effect created by elevated sugar concentrations (Rastogi et al., 2002), which reduces water activity and thereby inhibits the growth of spoilage microorganisms, including bacteria, yeasts, and molds. Lowering aw to levels below the minimum required for microbial proliferation is key to extending shelf life in high-sugar products (Maltini et al., 2003). Fruits used for candying include apple, date, papaya, cherry, plum, and pineapple, reflecting traditional preservation practices (Maltini et al., 2003; Sethi et al., 2025). These products are consumed as ready-to-eat snacks, incorporated into baked goods such as cakes and breads, and in some cultural contexts are believed to have health-promoting properties, adding to their market value. In addition to preservation, candying can reshape fruit quality by modifying sugar and organic acid composition, aroma expression, texture, and the retention or transformation of non-volatile compounds. Therefore, the chemical composition of the fresh raw material may continue to influence the quality of the final candied product under standardized candying conditions.

*Prunus mume* (*P. mume*), a perennial fruit crop of the Rosaceae family with an extensive cultivation history, is predominantly grown in East Asia, with China serving as the primary production hub. The major production regions include Sichuan, Zhejiang, Yunnan, Fujian, and Guangdong, each contributing distinctive ecotypes adapted to local agroclimatic conditions (Gong et al., 2021; Tian et al., 2023). *P. mume* fruit (PMF) constitutes a critical raw material for a diverse range of processed products, including candied preserves, Wumei (traditional smoked and desiccated plum), dried fruits, clarified and cloudy juices, fermented beverages (fruit vinegar and wine), as well as culinary condiments and flavoring agents (Sui et al., 2024). Beyond its culinary versatility and industrial applications, PMF is rich in bioactive phytochemicals, encompassing polyphenols, organic acids, flavonoids, and triterpenoids. These bioactive constituents confer a wide array of reported biological activities, including potent antioxidant capacity, prebiotic-mediated modulation of gut microbiota composition, and amelioration of intestinal inflammation and barrier dysfunction (Gong et al., 2021; Tian et al., 2023; Yang et al., 2024; Zhang et al., 2023). These functional attributes, coupled with its high acidity and characteristic volatile organic compound (VOC) composition, have positioned PMF as a valuable crop for both the food processing industry and emerging nutraceutical and functional food sectors. However, these desirable quality attributes are closely related to the chemical composition of harvested fruit, which may vary with geographical origin and local growing conditions.

Despite its high nutritional value and distinctive sour taste, PMF is a typical climacteric fruit, characterized by rapid ripening and pronounced susceptibility to postharvest decay (Gao et al., 2026). In the absence of appropriate postharvest interventions, PMF exhibits a limited shelf life, necessitating cold storage or ethylene management to preserve quality. This inherent perishability poses significant challenges for storage, transportation, and industrial scalability. To address these constraints, various processing techniques have been developed for PMF, including smoking to produce Wumei, drying, candying, juicing, and jam production (Hu et al., 2025; Sui et al., 2024). These processes not only mitigate the rapid spoilage of fresh fruit but also substantially enhance product value through intensive processing, thereby increasing farm-level profitability and promoting local economic development. Among these processed products, candied *P. mume* (CPM) is especially valued for its high-acid background combined with sugar enrichment during processing and its characteristic aromatic profile, making it a representative product within regional fruit-processing systems. Therefore, CPM provides a suitable product for comparing origin-related chemical quality differences when all samples are prepared under the same candying conditions (Luo et al., 2018). Previous studies on other *Prunus* species have shown that geographical origin and cultivar background can be reflected in different quality-related chemical dimensions (Li et al., 2022; Nie et al., 2023). For peach (*Prunus persica*), VOC profiling combined with chemometric analysis successfully discriminated fruits from different geographical origins (Giménez-Campillo et al., 2024). Metabolomic profiling of honey peaches from five main cultivation regions likewise revealed region-associated differences in metabolic profiles and identified potential discriminating metabolites (Li et al., 2022). In addition to these geographical effects, peach cultivars differ in aroma-related compounds, particularly esters, lactones, terpenoids, and aldehydes (Sun et al., 2022). For sweet cherry (*Prunus avium*), differences in physicochemical and selected phytochemical traits have been reported among production regions and cultivation locations, with these patterns associated with cultivar characteristics, climatic conditions, altitude, and soil properties (Nie et al., 2023). Cultivar-level studies have also revealed substantial variation in sugars, organic acids, phenolic compounds, anthocyanins, and antioxidant activity (Ballistreri et al., 2013; Usenik et al., 2008), while differences in sensory attributes among cultivars have additionally been reported (Usenik et al., 2008). Collectively, these studies indicate that geographical and cultivar-related quality differentiation within the genus *Prunus* can be reflected in aroma-related VOC_S_, taste-related compounds, and broader metabolic profiles. However, most previous studies have focused on fresh fruits and have generally examined geographical authentication, VOC composition, metabolite profiles, or physicochemical quality separately (Luykx & van Ruth, 2008). Whether origin-associated chemical differentiation can be detected after standardized processing, particularly in candied *P. mume*, remains insufficiently understood. Although previous studies on *P. mume* have investigated cultivar variation, phytochemical composition, antioxidant activity, or VOC profiles (Liu et al., 2022), integrated analyses linking physicochemical and functional traits, aroma-active compounds, VOC profiles, and non-volatile metabolites across multiple geographical origins of CPM remain limited. Therefore, this study did not aim simply to classify CPM samples by geographical origin, but to determine how origin-related chemical differences are reflected in CPM prepared under the same candying conditions. By integrating quality-trait assays, headspace solid-phase microextraction coupled with gas chromatography–mass spectrometry (HS-SPME-GC–MS)-based VOC profiling, untargeted metabolomics, relative odor activity value (ROAV) evaluation, weighted correlation network analysis (WCNA), random forest-Shapley additive explanations (RF-SHAP) analysis, and cross-layer correlation analysis, we aimed to identify the volatile and non-volatile chemical features associated with sugar–acid balance, antioxidant capacity, and aroma differences in candied *P. mume*. These results provide chemical evidence for understanding origin-related quality differences in CPM products.

## 2 Materials and methods

### 2.1 Fruit collection and candied processing

PMF were collected from fourteen representative origins across five major producing provinces in China during the same harvesting season (May–June 2025). Fruits at a comparable commercial maturity stage were selected, and damaged or diseased fruits were removed to ensure sample uniformity. For each origin, the collected fruits were randomly divided into three independent biological replicates for subsequent candied processing and analyses. The same maturity-screening criteria were applied to all origins: fruits were selected at the commercial green-mature stage, with no visible mechanical damage or disease symptoms, and their maturity status was further evaluated quantitatively using peel and flesh CIELAB color parameters and fruit firmness. Fruit developmental period was defined as the interval from full bloom to commercial harvest or maturity. The flowering and harvest/maturity periods of the corresponding production regions were used to characterize the phenological background of the fourteen origins, and the growth periods are summarized in **Table S1**. Before processing, peel and flesh color, as well as fruit firmness, were quantitatively evaluated using ten individual fruits from each origin (*n* = 10). Peel and flesh color were measured using an ST-700d Plus spectrophotometer (3nh, Shenzhen, China) and expressed as CIELAB L*, a*, and b* values. Fruit firmness was measured using a GY-3 fruit firmness tester equipped with a Φ8 mm probe and expressed as kg/cm^2^. The corresponding color and firmness data are provided in **Table S2**.

**Fig. 1:**
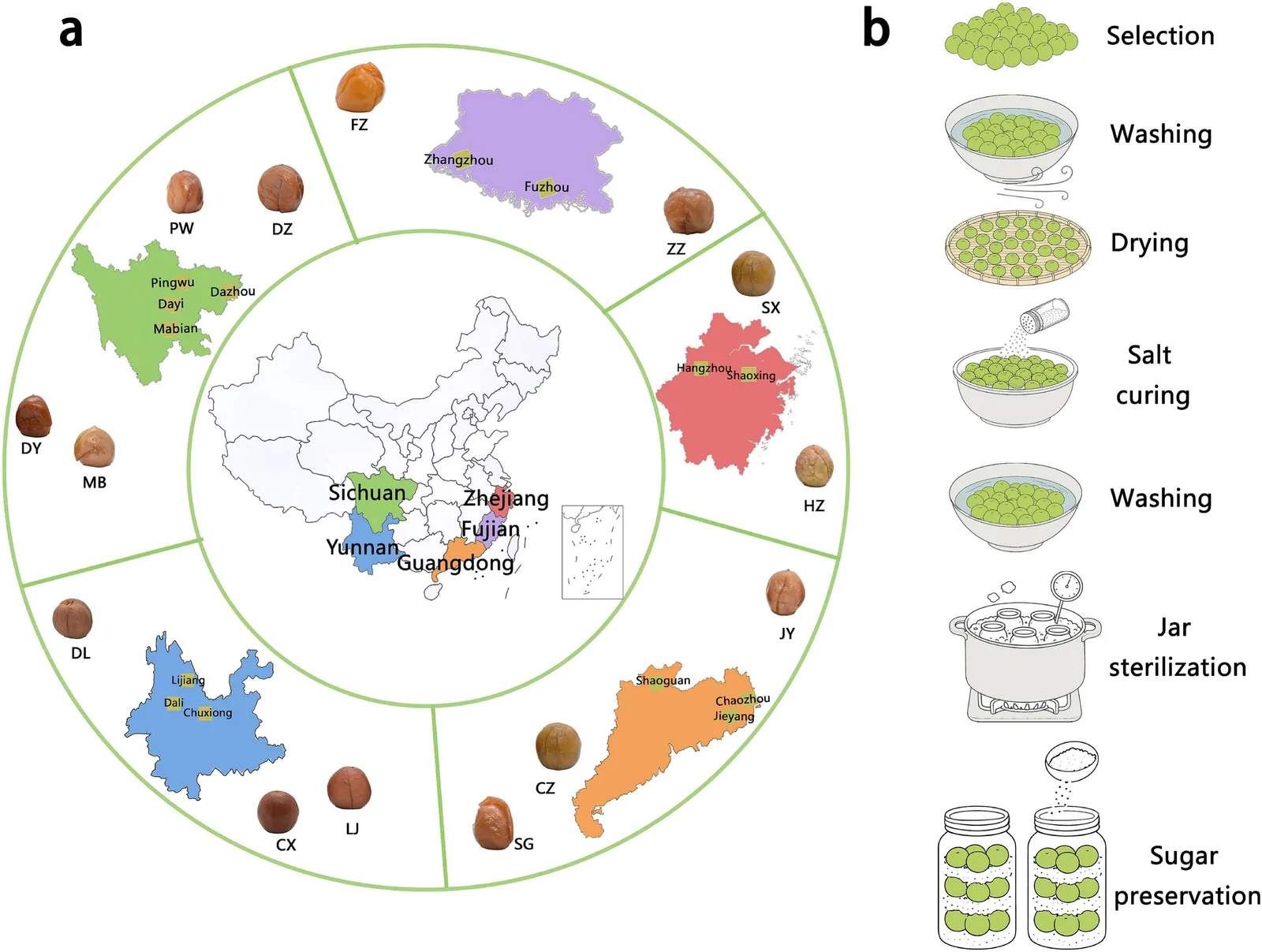
Geographical distribution of the fourteen origins of PMF in China and schematic illustration of the unified candied-processing procedure. (a) Sampling locations across five major producing provinces. (b) Standardized processing workflow, including collection, cleaning, salt curing, rinsing and drying, and sugar preservation. Three independent biological replicates were prepared and processed separately for each origin (*n* = 3).

The geographical distribution of the origins and the unified candied-processing workflow are shown in Fig. 1. After collection, samples were transported to the laboratory under identical conditions and processed promptly. Prior to candied processing, fruits were cured with salt at room temperature for 12 h, using salt at 30% (*w*/w) of the sugar amount. After salt curing, fruits were thoroughly washed with distilled water and air-dried. Candied processing was then conducted by mixing the fruits with granulated sugar at 50% (*w*/w) of the fruit weight and allowing the mixture to stand at room temperature for 5 d. All samples were processed under identical conditions without adjustment for geographical origin. Each biological replicate was processed separately throughout the experiment. After processing, samples from each replicate were independently homogenized before aliquots were taken for subsequent analyses. The use of three independent biological replicates was intended to represent within-origin biological variability, reduce the influence of random sampling variation, and provide independent observations for statistical analysis. The resulting CPM samples were collected and used for subsequent physicochemical analyses, VOC analysis, and metabolomic profiling.

### 2.2 Analysis of physicochemical traits and antioxidant activity

Soluble sugar content was determined by the anthrone colorimetric method (Xiao et al., 2026). Briefly, 0.2 g of the sample was extracted three times with 10 mL of 80% ethanol in a boiling water bath and centrifuged at 5000 rpm for 10 min. The combined supernatants were used for the determination of glucose, sucrose, and fructose under their respective reaction conditions. Absorbance was measured at 620 nm, and total soluble sugar content was calculated as the sum of sucrose, glucose, and fructose based on the corresponding standard curves. Total organic acid content was determined by acid–base titration. Briefly, 5 g of homogenized sample was extracted with 20 mL of distilled water at 80 °C for 30 min, and the clear extract was titrated with 0.5 mol/L NaOH using phenolphthalein as the indicator. Results were expressed as citric acid equivalents.

Total phenolic content (TPC) was determined by the Folin–Ciocalteu colorimetric method (Xiao et al., 2026) after ethanol extraction of the sample. Absorbance was measured at 765 nm, and TPC was calculated from a gallic acid standard curve. Total flavonoid content (TFC) was determined by the AlCl_3_ colorimetric method (Xiao et al., 2026) after ethanol extraction of the sample. Absorbance was measured at 510 nm, and TFC was calculated from a rutin standard curve.

ABTS radical scavenging activity was determined using the ABTS–potassium persulfate system, and absorbance was measured at 734 nm after reaction with the sample extract (Rumpf et al., 2023). DPPH radical scavenging activity was determined using the DPPH assay, with absorbance measured at 517 nm after incubation with the sample extract. Reducing power (RP) was determined using the potassium ferricyanide reduction method, and absorbance was measured at 700 nm. Vitamin C and Trolox were used as positive controls in all antioxidant assays (Shahidi & Samarasinghe, 2025).

### 2.3 HS-SPME-GC–MS analysis

VOCs from CPM samples collected from different geographical origins were determined by headspace solid-phase microextraction coupled with gas chromatography–mass spectrometry (HS-SPME-GC–MS). Analyses were carried out on a Shimadzu GC–MS-QP2020NX (Shimadzu, Kyoto, Japan) operated under electron impact ionization (EI, 70 eV) and fitted with an HP-5 capillary column (5% phenyl/95% dimethylpolysiloxane; 60 m × 0.25 mm i.d., 0.25 μm film) (Hu et al., 2025). Briefly, 0.20 g of CPM was placed in a 20 mL headspace vial containing 1.50 g of NaCl, 1.00 mL of deionized water, and 10 μL of methyl octanoate solution (0.04466 mg/mL) as the internal standard. The vial was pre-equilibrated at 60 °C with agitation at 250 rpm for 15 min. VOCs were then extracted at 60 °C for 30 min using a 50/30 μm DVB/CAR/PDMS fiber (Supelco, Bellefonte, PA, USA). After extraction, the fiber was immediately introduced into the GC inlet and thermally desorbed at 250 °C for 5 min. Helium (99.999%) served as the carrier gas at 1.0 mL/min. The oven program started at 50 °C, increased to 150 °C at 2 °C/min (held for 2 min), then ramped to 250 °C at 6 °C/min and held for 8 min. Mass spectra were acquired in the range of 33–600 *m*/*z* with the ion source maintained at 240 °C. VOC_S_ were tentatively identified by matching mass spectra to the NIST 2020 library, features with a similarity index ≥85% were tentatively assigned, whereas those with lower similarity were reported as unknowns. In addition, a C7–C30 n-alkane standard mixture was analyzed under identical conditions to calculate retention indices (RI), which were used to further support tentative compound annotation.

Semi-quantification of VOCs was performed using an internal standard approach, and VOC concentrations were estimated from the peak area ratio of each analyte to the internal standard together with the internal standard concentration, added volume, and sample weight. The results were expressed as μg/g. The odor activity value (OAV) was calculated as follows: OAV = C_i_ / T_i_, where C_i_ is the concentration of the VOC and T_i_ is its odor threshold. Compounds with OAV ≥ 1 were considered aroma-active compounds. The relative odor activity value (ROAV) was calculated according to the following equation: ROAV_i_ = (OAV_i_ / OAV_max_) × 100, where OAV_i_ is the odor activity value of a given compound and OAV_max_ is the highest OAV in the same sample. Compounds with ROAV ≥1 were considered key aroma contributors, whereas those with 0.1 ≤ ROAV <1 were regarded as potential modifiers of the overall aroma profile. Odor threshold values were obtained from published literature and public databases.

### 2.4 Analysis of non-volatile metabolites

#### 2.4.1 Sample preparation

Approximately 100 ± 5 mg of each CPM sample was accurately weighed into a 2 mL centrifuge tube, together with a 6 mm grinding bead. Subsequently, 400 μL of extraction solvent (methanol/water, 4:1, *v*/v), containing four internal standards, including L-2-chlorophenylalanine (0.02 mg/mL), was added before homogenization. Following the procedure described by Hu et al. (2025), the sample was homogenized directly in the extraction solvent using a cryogenic tissue grinder for 6 min at −10 °C and 50 Hz, thereby allowing sample disruption and initial metabolite extraction to occur simultaneously. The homogenates were then subjected to ultrasonic extraction at 40 kHz and 5 °C for 30 min, followed by protein precipitation at −20 °C for 30 min. After centrifugation at 13,000 ×*g* for 15 min at 4 °C, the supernatant was carefully collected and transferred into vials for instrumental analysis. To ensure analytical reliability, a pooled quality control (QC) sample was prepared by mixing 20 μL aliquots from each individual supernatant.

#### 2.4.2 UHPLC–MS/MS analysis

Non-volatile metabolite profiling was conducted using a UHPLC–Triple TOF-MS system (AB SCIEX, USA). Chromatographic separation was achieved on an ACQUITY UPLC HSS T3 column (100 mm × 2.1 mm i.d., 1.8 μm; Waters, Milford, USA). The mobile phases consisted of solvent A (water/acetonitrile, 95:5, *v*/v, with 0.1% formic acid) and solvent B (acetonitrile/isopropanol/water, 47.5:47.5:5, *v*/v/v, with 0.1% formic acid). The same gradient elution program was applied in both positive and negative ion modes as follows: 0–3.5 min, 2–50% B; 3.5–6.0 min, 50–95% B; 6.0–6.5 min, 95% B; 6.5–6.6 min, 95–2% B; and 6.6–8.0 min, 2% B for column re-equilibration. The flow rate was maintained at 0.40 mL/min, with an injection volume of 10 μL and a column temperature of 45 °C. Mass spectrometric data were acquired in both positive and negative ion modes over a mass range of *m*/*z* 50–1200. The curtain gas was set to 35 psi, while ion source gas 1 and gas 2 were both set at 50 psi, and the source temperature was maintained at 500 °C. The collision energies were set to 20, 40, and 60 eV, with ion spray voltages of 5.5 kV (positive mode) and − 4.5 kV (negative mode), respectively. QC samples, processed identically to study samples, were injected at regular intervals throughout the analytical sequence to assess system stability. Raw data were processed using Progenesis QI software (version 3.0, Waters Corporation, Milford, USA), including baseline correction, peak detection, alignment, retention time calibration, and normalization, resulting in a data matrix comprising retention time, *m*/*z* values, and peak intensities. Metabolite annotation was performed by matching MS and MS/MS spectra against relevant metabolite databases, with a mass error threshold below 10 ppm, and final identification was based on secondary mass spectral matching scores.

### 2.5 Random forest and SHAP-based feature prioritization

To prioritize non-volatile metabolites associated with origin-related metabolomic variation, a Random Forest (RF)-based workflow was applied to the metabolome dataset. An RF model was built using the full metabolite matrix to rank feature importance, and the top 100 RF-ranked metabolites were retained for subsequent SHAP-based interpretation. The RF model was run with 500 trees. SHA*P* values were calculated separately for the predicted output of each origin class using the fastshap package with 20 simulations, and mean absolute SHAP values were used to summarize metabolite importance at both class-specific and global levels. RF top-20 importance, global SHAP top-20 importance, and class-specific SHAP profiles were visualized as bar plots and beeswarm plots. Because the RF–SHAP workflow was used for feature prioritization and interpretation, the identified metabolites were considered candidate differentiating features rather than definitive origin markers. All analyses were performed in R using the packages randomForest, fastshap, shapviz, ggplot2, and dplyr, with a fixed random seed of 123 (Zheng et al., 2024).

### 2.6 Statistical analysis

Data from the CPM physicochemical, VOC, and non-volatile metabolite analyses were expressed as the mean ± standard deviation of three independent biological replicates (*n* = 3). Differences among samples from different geographical origins were evaluated using one-way analysis of variance (ANOVA) followed by Duncan's multiple range test, with statistical significance set at *P* < 0.05. Principal component analysis (PCA) was used to visualize overall variation in the VOC and non-volatile metabolite datasets. Partial least squares discriminant analysis (PLS-DA) was used as a supervised exploratory approach to assist feature screening, and variable importance in projection (VIP) values were used to rank discriminative variables. For inter-provincial comparisons of the non-volatile metabolite dataset, samples from all origins within each province were grouped by province and analyzed jointly in pairwise comparisons. Non-volatile metabolites with a false discovery rate (FDR) < 0.05 and a fold change (FC) ≥ 1.5 or ≤ 0.67 were considered differential, whereas differential VOCs were screened using FDR < 0.05 and |log_2_FC| ≥ 1. The results were visualized using volcano plots. Pearson correlation analysis was conducted to explore pairwise relationships between individual chemical variables and physicochemical or functional traits. For matrix-level integration, Mantel tests were used to assess the correspondence between distance matrices derived from the VOC, non-volatile metabolite, and quality-trait datasets. Compared with ordinary Pearson correlation, which evaluates associations between individual variable pairs, the Mantel test enables comparison of the overall similarity structure between multivariate datasets and is therefore suitable for assessing associations between high-dimensional omics profiles and quality-trait patterns (Peng et al., 2025; Yang et al., 2026). Statistical significance was determined by permutation testing at *P* < 0.05. Statistical analyses were performed using SPSS Statistics 26.0 (IBM Corp., Armonk, NY, USA), and figures were prepared using OriginPro 2022 (OriginLab Corp., Northampton, MA, USA). Multivariate analyses and related visualizations were additionally implemented in MetaboAnalyst 5.0 and R 4.2.2 (packages including ggplot2, ropls, and mixOmics).

## 3 Results and discussion

### 3.1 Variations in physicochemical and functional traits across origins

**Fig. 2:**
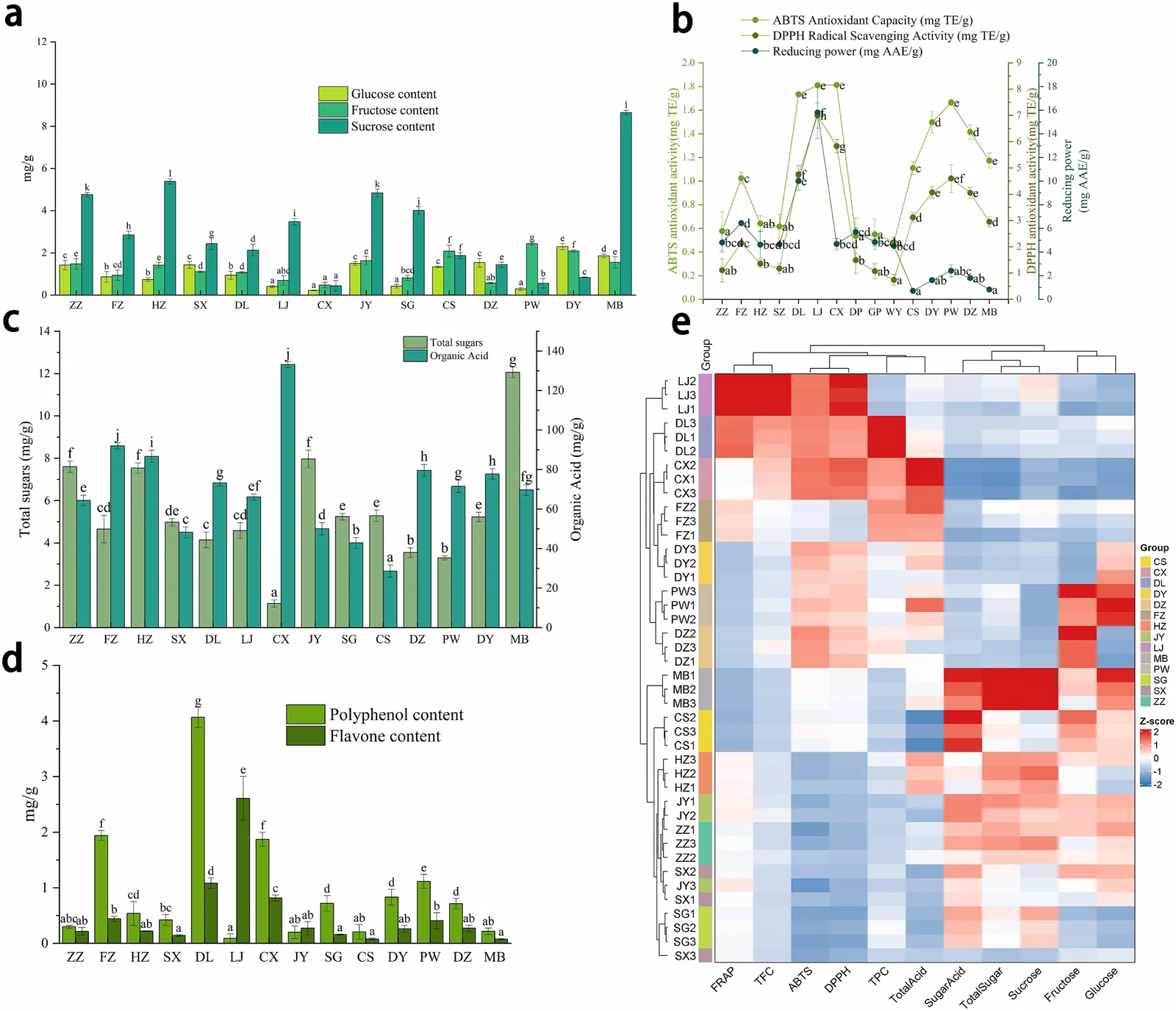
Comparison of quality-related traits among CPM samples from different origins. (a) Glucose, fructose, and sucrose contents. (b) Antioxidant capacity assessed by ABTS radical scavenging activity, DPPH radical scavenging activity, and RP. (c) Total soluble sugar content, total organic acid content, and sugar–acid ratio. (d) Total phenolic content (TPC) and total flavonoid content (TFC). (e) Heatmap and hierarchical clustering of quality-related traits across all samples, showing the overall differentiation pattern among origins. Data in panels (a–d) are expressed as the mean ± SD of three independent biological replicates (*n* = 3). Different lowercase letters in panels (a–d) indicate significant differences among origins according to one-way ANOVA followed by Duncan's multiple range test (*P* < 0.05). In panel (e), the data were *Z*-score normalized and subjected to hierarchical clustering.

Marked spatial variation was observed in the physicochemical and functional traits of CPM across the fourteen geographical origins (Fig. 2a–2e). Total soluble sugars, organic acids, and the sugar–acid ratio varied markedly among origins, indicating pronounced divergence in sugar–acid-related basic quality traits **(**Fig. 2a, c**)**. Functional indices also varied substantially, with TPC spanning 0.09–4.07, TFC 0.07–2.61, ABTS 0.49–1.81 mg TE/g, DPPH 0.74–6.82 mg TE/g, and RP 0.72–15.40 mg AAE/g, collectively suggesting strong origin-dependent heterogeneity in bioactive accumulation and antioxidant potential (Fig. 2b, d). MB exhibited the highest total soluble sugar level (12.06 ± 0.25 mg/g). In contrast, CX showed the strongest organic-acid-dominant chemical profile, with the highest organic acid level (13.31 ± 0.15 mg/g) but the lowest sugar content (1.14 ± 0.17 mg/g), resulting in the minimum sugar–acid ratio (0.09 ± 0.01). CZ showed the highest sugar–acid ratio (1.87 ± 0.26) supported by relatively higher sugars (5.28 ± 0.27 mg/g) and lower organic acid content (2.86 ± 0.31 mg/g). Functional quality traits further revealed clear stratification. DL displayed the highest phenolic accumulation (TPC: 4.07 ± 0.18) and strong flavonoid enrichment (TFC: 1.08 ± 0.10), accompanied by high antioxidant performance (RP: 10.40 ± 0.25 mg AAE/g). LJ showed the strongest radical-scavenging and reducing capacity (DPPH: 6.82 ± 0.20 mg TE/g, RP: 15.40 ± 0.14 mg AAE/g) together with the highest flavonoid level (TFC: 2.61 ± 0.40), indicating a distinctly function-oriented profile (Fig. 2b, d, e). This pattern is similar to prior work showing that phenolic profiles in *P. mume* are closely associated with antioxidant-related activities (Choi & Koh, 2017; Mitani et al., 2013) and can vary with origin (Liu et al., 2022). Together, these results indicate clear origin-related differences in basic quality traits among CPM samples under a unified candied-processing regime, with different origins showing distinct sugar and organic acid composition and functional profiles.

To provide an integrated interpretation of quality-related traits across origins, the fourteen CPM origins could be broadly characterized according to their combined sugar–acid balance, phenolic accumulation, and antioxidant capacity profiles (Fig. 2e). MB exhibited a relatively sweet-oriented profile due to its high soluble sugar content, which may favor processing strategies that emphasize sweetness. In contrast, CX showed an acid-dominant profile with the highest organic acid content and the lowest sugar–acid ratio, indicating a stronger sour flavor tendency that may be relevant to products emphasizing pronounced acidity or requiring additional sweetness for flavor balance. CZ displayed the highest sugar–acid ratio, indicating a relatively less acid-dominant sweet–sour chemical profile among the examined origins. DL and LJ showed higher phenolic/flavonoid accumulation and stronger antioxidant-related indices, indicating relatively prominent antioxidant-related chemical characteristics among the examined origins. Other origins exhibited intermediate or mixed characteristics, reflecting diverse quality combinations rather than a single optimal profile. These findings suggest that origin selection could be considered according to the intended processing objective, with sweet-oriented, acid-rich, and antioxidant-enriched origins showing different potential advantages for product development.

### 3.2 Origin differentiation of volatile organic compounds (VOC_S_)

#### 3.2.1 Overall VOC profiles among fourteen origins

**Fig. 3:**
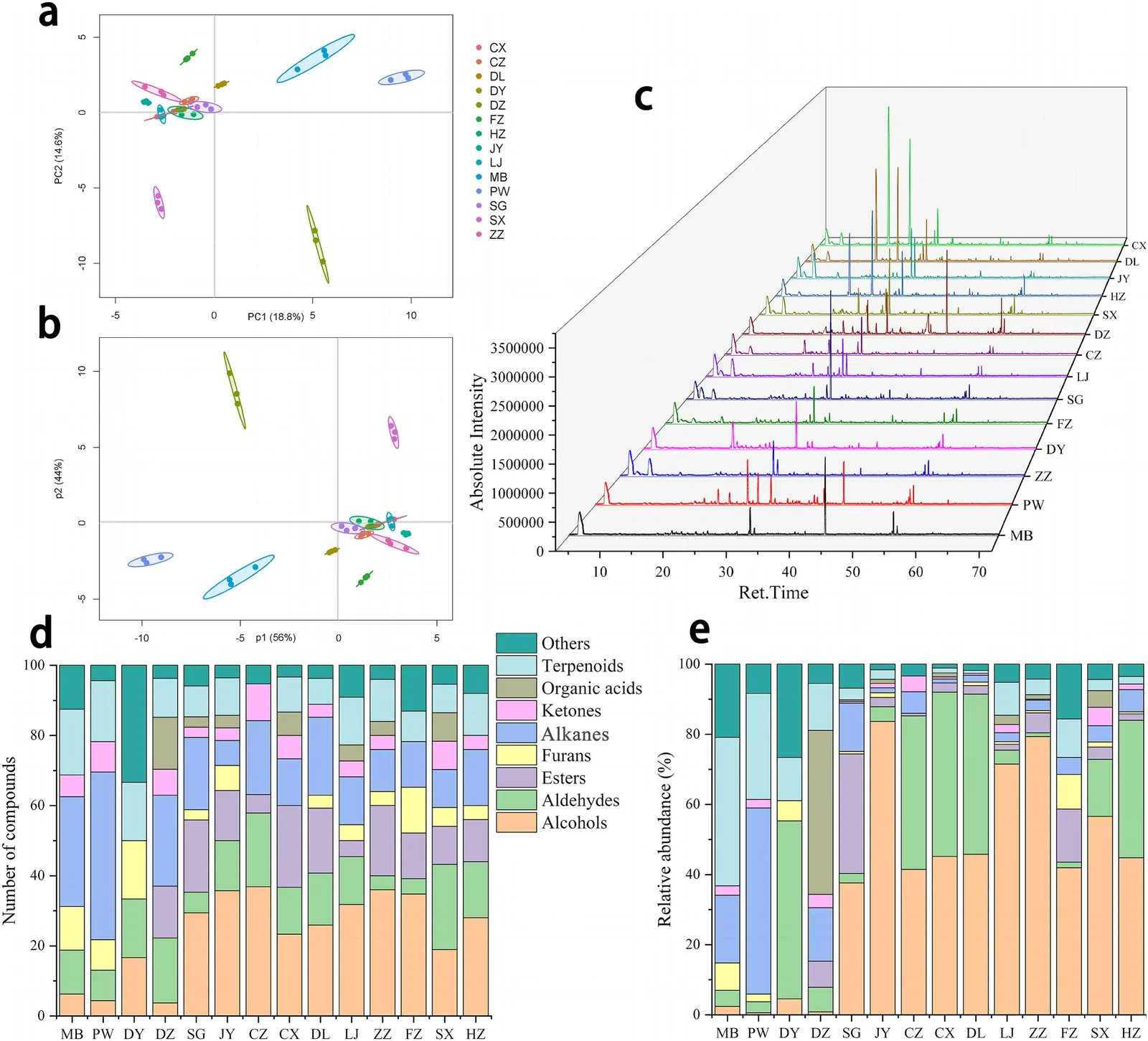
Overall VOC profiles and chemical composition of CPM across fourteen origins. (a) PCA score plot of VOC profiles. (b) PLS-DA score plot showing the supervised distribution of samples among origins. (c) Representative GC–MS total ion chromatograms (TICs). (d) Percentage composition of VOC chemical classes based on compound counts. (e) Percentage composition of the nine major VOC classes based on their summed relative abundances. Each origin included three independent biological replicates (*n* = 3). Ellipses in panels (a) and (b) represent 95% confidence intervals.

GC–MS profiling showed that the VOC composition of CPM varied markedly among geographical origins in terms of overall profile distribution and chemical-class composition (Fig. 3a–3e, Table 1). The PCA and PLS-DA score plots showed visible origin-related separation in VOC profiles, and representative GC–MS total ion chromatograms further illustrated differences in peak distribution among origins (Fig. 3a–3c). In total, 75 distinct VOCs were tentatively identified across all samples and classified into nine chemical classes, including alcohols, aldehydes, ketones, esters, furans, alkanes, acids, terpenoids, and others (Table 1**, Table S3**). For each origin, the total number of distinct VOCs, calculated across all nine chemical classes, ranged from 6 to 37 (Fig. 3d, Table 1), indicating substantial variation in VOC richness among origins.

**Table 1:** Tentative identification information of representative volatile organic compounds (VOCs) in candied *Prunus mume* (CPM).

Compounds	Substance class	CAS	RI
2-Ethyl-1-hexanol	Alcohols	104–76-7	1028
1-Nonanol	Alcohols	143–08-8	1171
1-Octanol	Alcohols	111–87-5	1068
1-Octen-3-ol	Alcohols	3391-86-4	981
(*Z*)-2-Octen-1-ol	Alcohols	26,001–58-1	1066
Benzyl alcohol	Alcohols	100–51-6	1037
Phenylethyl alcohol	Alcohols	60–12-8	1116
3-Methyl-1-butanol	Alcohols	123–51-3	730
1-Heptanol	Alcohols	111–70-6	970
1-Hexanol	Alcohols	111–27-3	867
2-Methyl-1-propanol	Alcohols	78–83-1	626
7-Octen-2-ol, 2-methyl-6-methylene-	Alcohols	543–39-5	1124
1,5,7-Octatrien-3-ol, 3,7-dimethyl-	Alcohols	29,957–43-5	1104
α-Terpineol	Alcohols	98–55-5	1192
Linalool	Alcohols	78–70-6	1097
trans-Ocimenol	Alcohols	7643-60-9	1168
(E,E)-2,4-Heptadienal	Aldehydes	4313-03-5	1017
Benzaldehyde	Aldehydes	100–52-7	964
2,4-Dimethylbenzaldehyde	Aldehydes	15,764–16-6	1180
Nonanal	Aldehydes	124–19-6	1087
(E)-2-Heptenal	Aldehydes	18,829–55-5	958
(E)-2-Nonenal	Aldehydes	18,829–56-6	1162
(E)-2-Octenal	Aldehydes	2548-87-0	1064
Citral	Aldehydes	5392-40-5	1276
Hexanal	Aldehydes	66–25-1	801
Octanal	Aldehydes	124–13-0	1007
Methyl benzoate	Esters	93–58-3	1096
Ethyl benzoate	Esters	93–89-0	1173
Ethyl decanoate	Esters	110–38-3	1396
Ethyl dodecanoate	Esters	106–33-2	1597
Nonyl acetate	Esters	143–13-5	1314
Ethyl acetate	Esters	141–78-6	614
Ethyl octanoate	Esters	106–32-1	1195
3-Hydroxy-2,2,4-trimethylpentyl isobutyrate	Esters	77–68-9	1380
2-Phenylethyl acetate	Esters	103–45-7	1257
Benzyl acetate	Esters	140–11-4	1162
Ethyl hexadecanoate	Esters	628–97-7	1994
Methyl salicylate	Esters	119–36-8	1193
Ethyl hexanoate	Esters	123–66-0	1002
Hexyl acetate	Esters	142–92-7	1011
Butyl hexanoate	Esters	626–82-4	1186
Hexyl hexanoate	Esters	6378-65-0	1385
2,2,5,5-Tetramethyltetrahydrofuran	Furans	15,045–43-9	759
2(3H)-Furanone, 5-hexyldihydro-	Furans	706–14-9	1470
2-Butyltetrahydrofuran	Furans	1004-29-1	1026
4-Methyldodecane	Alkanes	6117-97-1	1260
Hexadecane	Alkanes	544–76-3	1590
Tetradecane	Alkanes	629–59-4	1410
5-Methyltetradecane	Alkanes	25,117–32-2	1454
4,6-Dimethyldodecane	Alkanes	61,141–72-8	1325
Heptadecane	Alkanes	629–78-7	1700
Pentadecane	Alkanes	629–62-9	1502
4-Methyltetradecane	Alkanes	25,117–24-2	1457
3,7-Dimethyldecane	Alkanes	17,312–54-8	1127
2,5-Dimethylundecane	Alkanes	17,301–22-3	1210
Dodecane	Alkanes	112–40-3	1190
5-Butylnonane	Alkanes	17,312–63-9	1204
Pseudoionone	Ketones	689–67-8	1456
6-Methyl-5-hepten-2-one	Ketones	110–93-0	985
β-Damascenone	Ketones	23,726–93-4	1386
1-Octen-3-one	Ketones	4312-99-6	980
2-Methylbutanoic acid	Acids	116–53-0	886
Octanoic acid	Acids	124–07-2	1178
Benzoic acid	Acids	65–85-0	1180
Hexanoic acid	Acids	142–62-1	981
Nonanoic acid	Acids	112–05-0	1280
2,4-Di-tert-butylphenol	Others	96–76-4	1519
(1-Butylhexyl)benzene	Others	4537-11-5	1535
N,N-Dibutylformamide	Others	761–65-9	1319
Geranic oxide	Terpenoids	7392-19-0	972
trans-Linalool oxide (furanoid)	Terpenoids	34,995–77-2	1091
Ocimene quintoxide	Terpenoids	7416-35-5	1045
(Z)-Linalool oxide (furanoid)	Terpenoids	5989-33-3	1075
α-Pinene	Terpenoids	80–56-8	938
β-Myrcene	Terpenoids	123–35-3	992

Notes: “−” = not detected. CAS, Chemical Abstracts Service registry number; RI, retention index. RI values were calculated using a C7–C30 n-alkane series under the same chromatographic conditions and compared with values in the National Institute of Standards and Technology (NIST) database.

At the provincial level, Sichuan (MB, PW, DY, and DZ) exhibited a mixed VOC profile, without a single overwhelmingly dominant class, instead, terpenoids (26.68%), acids (20.66%), and alkanes (20.23%) contributed most to the overall VOC composition. By contrast, Guangdong (JY, SG, and CZ) and Fujian (ZZ and FZ) were both clearly dominated by alcohols, which accounted for approximately 67.17% and 66.79% of the total VOC abundance, respectively, whereas esters remained minor components. Their geographical proximity and broadly comparable warm and humid conditions during May–June 2025 may have partly contributed to their similar alcohol-dominated VOC profiles (**Table S1**). Differences in local cultivar or germplasm composition may also have contributed to the observed VOC patterns, together with regional environmental conditions. Yunnan (CX, DL, and LJ) was characterized by a VOC profile dominated by alcohols (52.21%) and aldehydes (35.32%), while Zhejiang (SX and HZ) showed a broadly similar class-level pattern, with alcohols and aldehydes together accounting for more than 80% of the total VOC abundance. Nevertheless, the two provinces differed markedly in their environmental conditions. Zhejiang experienced relatively uniform warm and humid conditions during May–June 2025, with mean temperatures of 23.66–23.78 °C and cumulative precipitation of 537.63–578.48 mm, whereas the Yunnan origins were generally cooler and more climatically heterogeneous, with mean temperatures ranging from 16.10 to 21.77 °C and cumulative precipitation from 387.10 to 512.03 mm (**Table S1**). These environmental differences may influence the accumulation of fatty-acid-, amino-acid-, and phenylalanine-derived volatile precursors, as well as the metabolic balance among aldehyde formation, aldehyde reduction, alcohol accumulation, and esterification. Differences in local cultivar or germplasm backgrounds may also partly explain the compound-level VOC differences observed between Zhejiang and Yunnan.

**Fig. 4:**
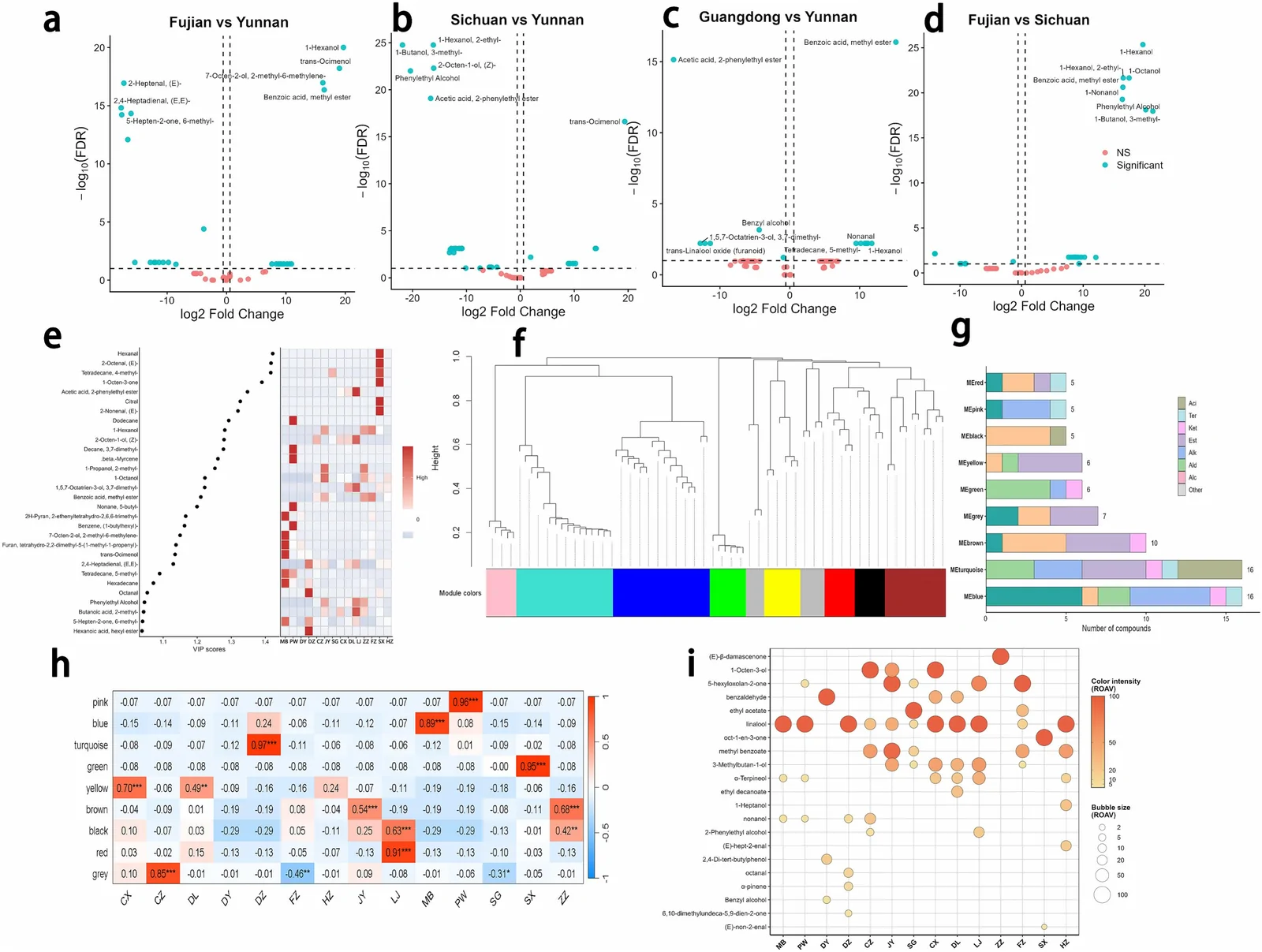
Differential screening, odor-activity evaluation, and WCNA-based module characterization of VOCs in candied CPM across origins. (a-d) Volcano plots of differential VOCs in representative provincial comparisons. Differential VOCs were screened using FDR < 0.05 and |log₂FC| ≥ 1. (e) VIP ranking of key discriminant VOCs and their abundance heatmap across the fourteen origins. (f) Hierarchical clustering dendrogram of WCNA-derived VOC modules. (g) Chemical-class composition of the identified VOC modules. (h) Pearson correlation heatmap between VOC modules and origins. Values in the cells represent Pearson correlation coefficients. Asterisks indicate statistical significance: *, *P* < 0.05. **, *P* < 0.01. and ***, *P* < 0.001. (i) Bubble plot of major odor-active VOCs based on relative odor activity values (ROAVs). Bubble size and color indicate ROAV magnitude, as shown in the legends. Each origin included three independent biological replicates (*n* = 3).

Importantly, although Zhejiang and Yunnan showed similar overall proportions of alcohols and aldehydes, they differed in the identities and relative abundances of individual VOCs, as illustrated by the hierarchical clustering heatmap (**Fig. S3**). Because individual VOCs differ substantially in odor thresholds and aroma characteristics (Sun et al., 2022), similar chemical-class proportions do not necessarily result in similar potential aroma profiles. The subsequent ROAV analysis further showed that Zhejiang and Yunnan differed in their dominant odor-active contributors (Fig. 4i). Therefore, their different potential aroma expressions may be associated with compound-level VOC composition and odor potency rather than with the summed proportions of alcohols and aldehydes alone. Broad provincial patterns masked pronounced within-province divergence, indicating clear VOC differentiation among origins from the same province (Wang et al., 2022; Zhao et al., 2025). Within Sichuan, VOC composition varied markedly among origins. MB was characterized by terpene-related VOC_S_, especially linalool, trans-ocimenol, and geranic oxide, whereas PW showed a terpene- and alkane-associated profile represented by linalool, 3,7-dimethyldecane, and 4,6-dimethyldodecane. DY showed a benzaldehyde-associated aldehyde profile, while DZ was distinguished by acid-related VOC_S_ such as nonanoic acid, hexanoic acid, and octanoic acid. Overall, these contrasts show that the VOC profile of Sichuan CPM is not uniform, but instead reflects multiple origin-specific compositional patterns within the same province (Fig. 3e). A similar within-province heterogeneity was also observed in Guangdong, where the three origins showed distinct VOC compositional patterns despite being from the same province. JY was strongly dominated by alcohols (83.67%), mainly represented by 3-methyl-1-butanol, phenylethyl alcohol, 2-methyl-1-propanol, and 1-hexanol. SG showed relatively high contributions from both alcohols (37.60%) and esters (33.81%), with 3-methyl-1-butanol and phenylethyl alcohol representing the alcohol fraction and ethyl acetate representing the ester fraction. CZ exhibited a mixed profile characterized by both aldehydes (43.27%) and alcohols (42.15%), mainly represented by benzaldehyde, benzyl alcohol, phenylethyl alcohol, and 1-octen-3-ol. These contrasts indicate that, even within the same province, origin-level VOC differentiation was reflected not only in chemical-class proportions, but also in different dominant representative VOCs (Fig. 3e). Within Fujian, both origins remained alcohol-rich, but their representative VOC profiles differed. ZZ was mainly characterized by alcohols such as 3-methyl-1-butanol, phenylethyl alcohol, 2-methyl-1-propanol, and 1-hexanol, whereas FZ showed a more mixed profile involving alcohols, esters, and terpene-related VOC_S_, represented by 3-methyl-1-butanol, 1-hexanol, ethyl acetate, trans-ocimenol, and geranic oxide. In Yunnan, alcohols and aldehydes remained co-dominant overall, although their relative contributions varied among geographical origins. CX and DL showed a pronounced benzenoid aldehyde/alcohol signature, mainly represented by benzaldehyde and benzyl alcohol, whereas LJ exhibited a more alcohol-dominant profile, represented by 3-methyl-1-butanol and phenylethyl alcohol (Ma et al., 2025; Zhao et al., 2025). Zhejiang also showed clear origin-specific restructuring despite its relatively balanced provincial profile, with SX exhibiting a higher alcohol contribution (56.57%), mainly represented by 3-methyl-1-butanol, benzyl alcohol, and phenylethyl alcohol. In contrast, HZ showed a stronger aldehyde contribution (39.24%), mainly driven by benzaldehyde, together with a high level of benzyl alcohol. These compound-level differences further explain why Zhejiang and Yunnan could share similar broad alcohol–aldehyde proportions but exhibit different potential aroma profiles. VOC differentiation showed a hierarchical pattern: province-level tendencies described broad VOC-class backgrounds, whereas origin-level differences revealed finer compound-level compositional features. Subsequent analyses therefore focused on discriminative VOCs, co-abundance modules, and potential key odor-active contributors.

#### 3.2.2 Multivariate analysis and key differential VOCs

Candidate VOCs contributing to origin discrimination were screened using PLS-DA-based VIP ranking together with differential abundance analysis (Fig. 4a–4e). VIP ranking highlighted a focused set of discriminant VOCs spanning multiple chemical classes, with hexanal (VIP = 1.422), (E)-2-octenal (VIP = 1.417), and 4-methyltetradecane (VIP = 1.416) ranking among the leading contributors (Fig. 4e). The remaining VIP-ranked VOCs also covered alcohols, aldehydes, ketones, esters, terpenoid-related compounds, alkanes, and acids. Therefore, VOC-based origin discrimination was not attributable to one dominant compound family, but to the combined contribution of origin-preferential aldehyde-type VOCs and broader abundance shifts across alcohols, esters, terpenoid-related compounds, alkanes, and volatile acids. Differential analysis further showed that VOC composition was restructured in an origin-dependent manner (Fig. 4a-4d). Using the thresholds of FDR < 0.05 and |log2FC| ≥ 1, each representative comparison yielded a distinct set of significantly altered VOCs, with the largest shift observed for Fujian vs Yunnan (28 VOCs) and a more moderate but still clear difference for Fujian vs Sichuan (20 VOCs). The labeled differential VOCs differed markedly among comparisons. Fujian vs Yunnan was mainly associated with changes in alcohols, terpenoid-related compounds, esters, aldehydes, and ketones, represented by 1-hexanol, trans-ocimenol, benzoic acid methyl ester, (E)-2-heptenal, (E,E)-2,4-heptadienal, and 6-methyl-5-hepten-2-one. Sichuan vs Yunnan involved alcohol- and ester-related VOCs such as 2-ethyl-1-hexanol, 3-methyl-1-butanol, phenylethyl alcohol, (Z)-2-octen-1-ol, and 2-phenylethyl acetate. Guangdong vs Yunnan was characterized by benzoic acid methyl ester, 2-phenylethyl acetate, benzyl alcohol, nonanal, 1-hexanol, tetradecane, and trans-linalool oxide (furanoid), whereas Fujian vs Sichuan mainly involved alcohols and benzoic acid methyl ester. Together, these results indicate that inter-provincial VOC differentiation was comparison-specific and involved multiple chemical classes rather than a single shared differential VOC pattern across all comparisons (Costa et al., 2024).

#### 3.2.3 Co-abundance modules and origin-associated VOC organization (WCNA)

As shown in Fig. 4f–4h, to further determine whether origin-related VOC differentiation was organized at the module level rather than only at the level of individual compounds, we applied WCNA, a network-based approach that groups VOCs showing similar abundance patterns across samples, to the VOC dataset (Zhang & Horvath, 2005). Based on the correlation structure across samples, the 75 VOCs were clustered into eight co-abundance modules (MEblue, MEturquoise, MEbrown, MEgreen, MEyellow, MEblack, MEpink, MEred) plus a small MEgrey set of unassigned features. Module sizes ranged from 5 to 16 compounds, indicating that origin-related VOC variation was represented by coordinated multi-compound patterns rather than scattered individual features. Detailed information on all 75 VOCs, including their compound names, chemical classes, and corresponding WCNA module assignments, is provided in Supplementary **Table S4**. Importantly, the WCNA module-origin correlation matrix (Fig. 4h) showed that only some origins exhibited a clear and statistically supported dominant module association, whereas others showed weaker or less concentrated associations across modules. Strong one-to-one links were observed for PW–MEpink (*r* = 0.96), DZ–MEturquoise (*r* = 0.97), MB–MEblue (*r* = 0.89), LJ–MEred (*r* = 0.91), SX–MEgreen (*r* = 0.95), and CZ–MEgrey (*r* = 0.85) (Fig. 4h), indicating that these origins were characterized by highly concentrated co-abundance patterns. In comparison, the remaining origins did not exhibit equally strong dominant-module relationships, suggesting comparatively weaker or less concentrated module associations. Overall, VOC organization differed mainly in the degree of module concentration, with some origins showing stronger associations with specific VOC modules, whereas others exhibited weaker or less concentrated module associations.

The chemical class composition of these modules further supported their chemical specialization (Fig. 4g). The PW-associated MEpink module was enriched in alkanes and terpenoids, indicating a module dominated by these compound classes. The DZ-associated MEturquoise module showed a mixed chemical composition spanning acids, esters, aldehydes, alkanes, and terpenoids, suggesting coordinated remodeling rather than a shift in a single chemical class. The SX-associated MEgreen was biased toward aldehydes, indicating an aldehyde-rich module. In contrast, MEyellow was enriched in esters, whereas MEbrown was characterized by a combined alcohol and ester composition. Together, these results indicate that VOC differentiation among origins in CPM was reflected not only at the level of individual compounds, but also in coordinated co-abundance patterns. VOCs were organized into compositionally distinct modules, and different origins showed different module association patterns, supporting the observed VOC differentiation across the dataset.

#### 3.2.4 ROAV assessment of key odorants

To estimate the potential aroma contribution of VOC variation among the fourteen geographical origins, ROAV analysis, which ranks VOC_S_ according to their relative odor contribution within each sample, was performed for all detected VOC_S_ (Fig. 4i), with compounds showing ROAV ≥1 defined as potential key odor-active contributors (Wu et al., 2025). The corresponding OAV values of the identified VOCs across the fourteen origins are provided in **Table S5**. The results indicated that the odor-active profiles of CPM were shaped by both shared core odorants and dominant contributors from specific origins. Among them, linalool was the most consistently detected key odorant and ranked first in several origins, including MB, PW, DZ, CX, DL, LJ, and HZ, indicating that it represented a recurrent odor-active component across these samples (Fig. 4i). Linalool has been previously associated with floral, citrus, and sweet odor characteristics (Sun et al., 2022). Other recurrent contributors, including α-terpineol, 2-phenylethyl alcohol, and 3-methyl-1-butanol, were also identified as key odorants in multiple origins, although their relative importance varied markedly among geographical origins. In contrast, several compounds were clearly dominant at specific origins and therefore contributed strongly to VOC-based aroma differentiation among origins. Benzaldehyde was the dominant odor-active compound in DY, and also showed high ROAV values in CX, DL, and HZ, suggesting that benzaldehyde contributed to the origin-dependent odor-active profiles of several samples. 1-octen-3-ol was particularly prominent in CZ and CX and also showed a high ROAV value in JY. Ethyl acetate showed the highest odor contribution in SG, while (*E*)-β-damascenone and 1-octen-3-one were identified as characteristic dominant odorants in ZZ and SX, respectively. Methyl benzoate also showed high ROAV values in JY, CZ, FZ, and HZ, indicating that benzenoid ester-type VOCs contributed to the odor-active profiles of multiple origins.

Compared with these relatively concentrated odor-active patterns, JY, CX, DL, and LJ exhibited more complex ROAV-based aroma profiles, in which multiple compounds simultaneously showed high ROAV values, suggesting a more diversified odor-active profile involving multiple aroma-related VOCs. For example, JY showed high ROAV values for methyl benzoate, 1-octen-3-ol, 3-methyl-1-butanol, linalool, and 5-hexyloxolan-2-one, whereas CX and DL combined high ROAV values of linalool, benzaldehyde, α-terpineol, benzyl alcohol, ethyl decanoate, and 2-phenylethyl alcohol. LJ was characterized by high ROAV values of linalool, 3-methyl-1-butanol, α-terpineol, 2-phenylethyl alcohol, and 5-hexyloxolan-2-one.

Overall, ROAV analysis suggested that origin-related aroma divergence in CPM was associated with the relative odor contributions of key VOCs, rather than VOC abundance alone (Fig. 4i). A conserved set of shared contributors provided the basic common aroma-active compounds, whereas a limited number of high-impact local odorants further differentiated the ROAV-based aroma profiles of individual geographical origins. When considered together with the WCNA-derived VOC modules, these ROAV patterns suggest that CPM VOC-based aroma differentiation involved both high-impact odorants and broader coordinated VOC organization.

### 3.3 Non-volatile metabolite differentiation across origins

#### 3.3.1 Spatial metabolic differentiation across geographical origins

**Fig. 5:**
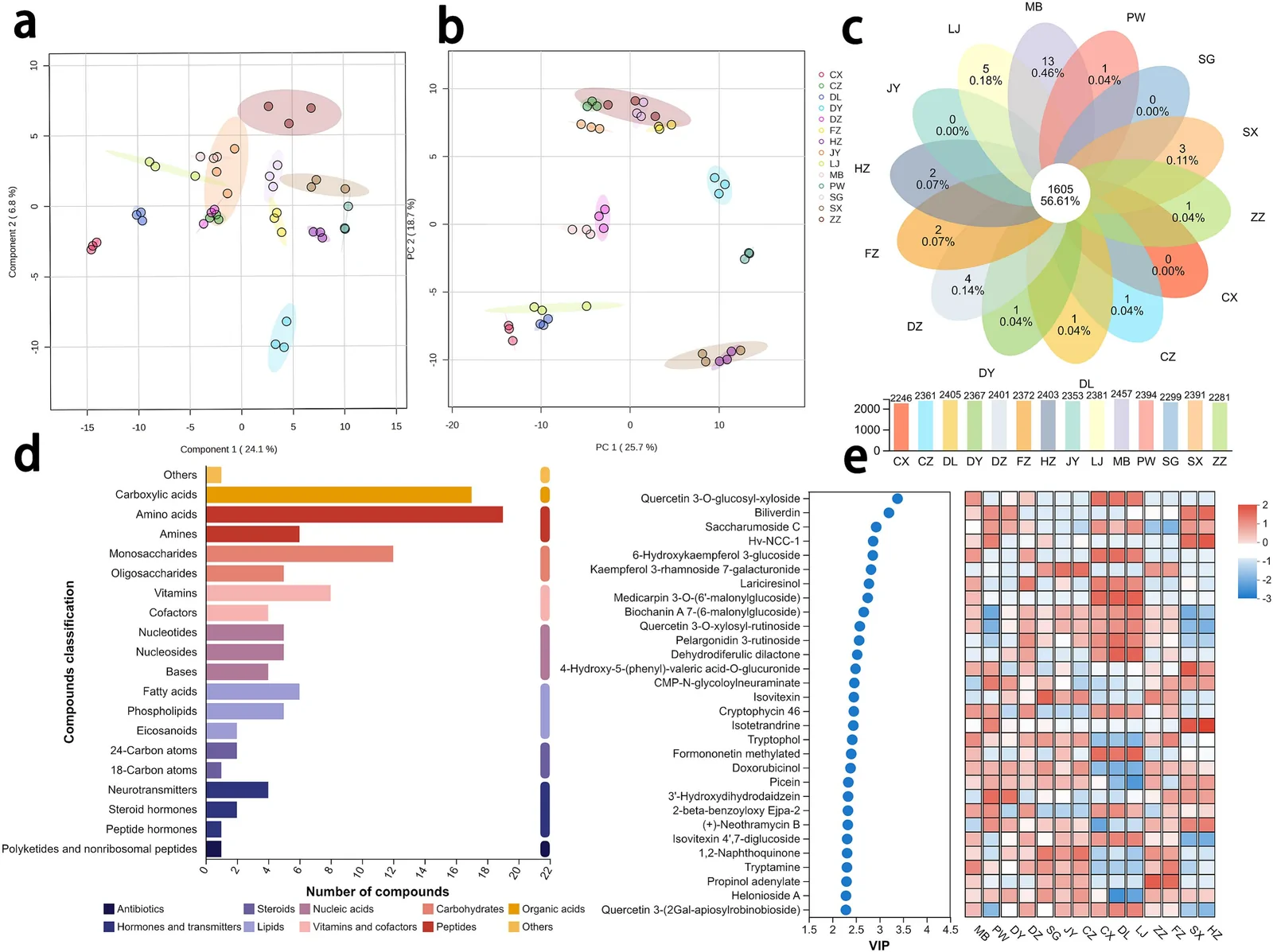
Spatial metabolic differentiation and compositional characteristics of the non-volatile metabolites in CPM across the fourteen origins. (a) PCA score plot of non-volatile metabolite profiles. (b) PLS-DA score plot showing the supervised distribution of samples among origins. (c) Distribution of shared and origin-specific metabolites, with total metabolite numbers for each origin shown below. (d) Classification of detected metabolites into major chemical categories. (e) VIP ranking of representative metabolites and their abundance heatmap across origins. Each origin included three independent biological replicates (*n* = 3). Metabolites with VIP > 1 were considered influential variables contributing to origin differentiation.

The non-volatile metabolite profile of CPM showed pronounced differences across the fourteen origins, indicating origin-dependent metabolomic differentiation under a unified candied-processing regime. In the PCA score plot, PC1 and PC2 explained 24.1% and 8.8% of the total variance, respectively (Fig. 5a). Samples from the same origin clustered closely, whereas those from different origins were clearly separated. PLS-DA showed a supervised separation pattern broadly consistent with the PCA-based pattern (Fig. 5b), with the first two components explaining 25.7% and 18.7% of the variation, respectively. Additional overviews of shared metabolites, compound classes, and representative discriminative features are shown in Fig. 5c-5e. The non-volatile metabolite dataset covered multiple biochemical categories, with amino acids, carboxylic acids, monosaccharides, oligosaccharides, vitamins/cofactors, nucleotides/nucleosides, fatty acids, phospholipids, and several hormone- or peptide-related compounds forming the main chemical background of CPM (Fig. 5d). This broad class distribution suggested that origin-related metabolomic variation involved both primary metabolic pools and specialized or regulatory metabolites. Meanwhile, this origin-level divergence occurred against a backdrop of largely conserved metabolites. A total of 1605 metabolites (56.61%) were shared across all origins, whereas origin-specific metabolites were limited, ranging from 0 to 13 per origin. The numbers of detected metabolites were similar across origins (2281–2457 per origin), suggesting that the inter-origin separation was more likely related to differences in metabolite abundance patterns than to metabolite number alone (Fig. 5c). Collectively, Fig. 5c indicates that the non-volatile metabolite repertoire of CPM was largely conserved across origins, allowing subsequent VIP and differential analyses to focus on abundance-based discriminative features within this shared metabolite background.

To further examine the chemical features associated with separation among origins, VIP ranking and differential feature screening were performed. VIP analysis identified 416 metabolites with VIP > 1, suggesting that separation among origins was associated with a broad set of influential metabolites rather than only a few dominant features. The highest-ranked contributors included Quercetin 3-O-glucosyl-xyloside, Biliverdin, Saccharumoside C, Hv-NCC-1, 6-Hydroxykaempferol 3-glucoside, and Kaempferol 3-rhamnoside 7-galacturonide, followed by lariciresinol, medicarpin 3-O-(6′-malonylglucoside), Biochanin A 7-(6-malonylglucoside), and Quercetin 3-O-xylosyl-rutinoside (Fig. 5e). These top VIP-ranked metabolites mainly included bioactive functional metabolites such as flavonoid glycosides, lignans, isoflavonoid glycosides, and other glycoside-type metabolites, together with tetrapyrrole/chlorophyll-catabolite-related pigments, indicating that metabolomic differentiation among origins was not confined to a single chemical group (Fig. 5e).

**Fig. 6:**
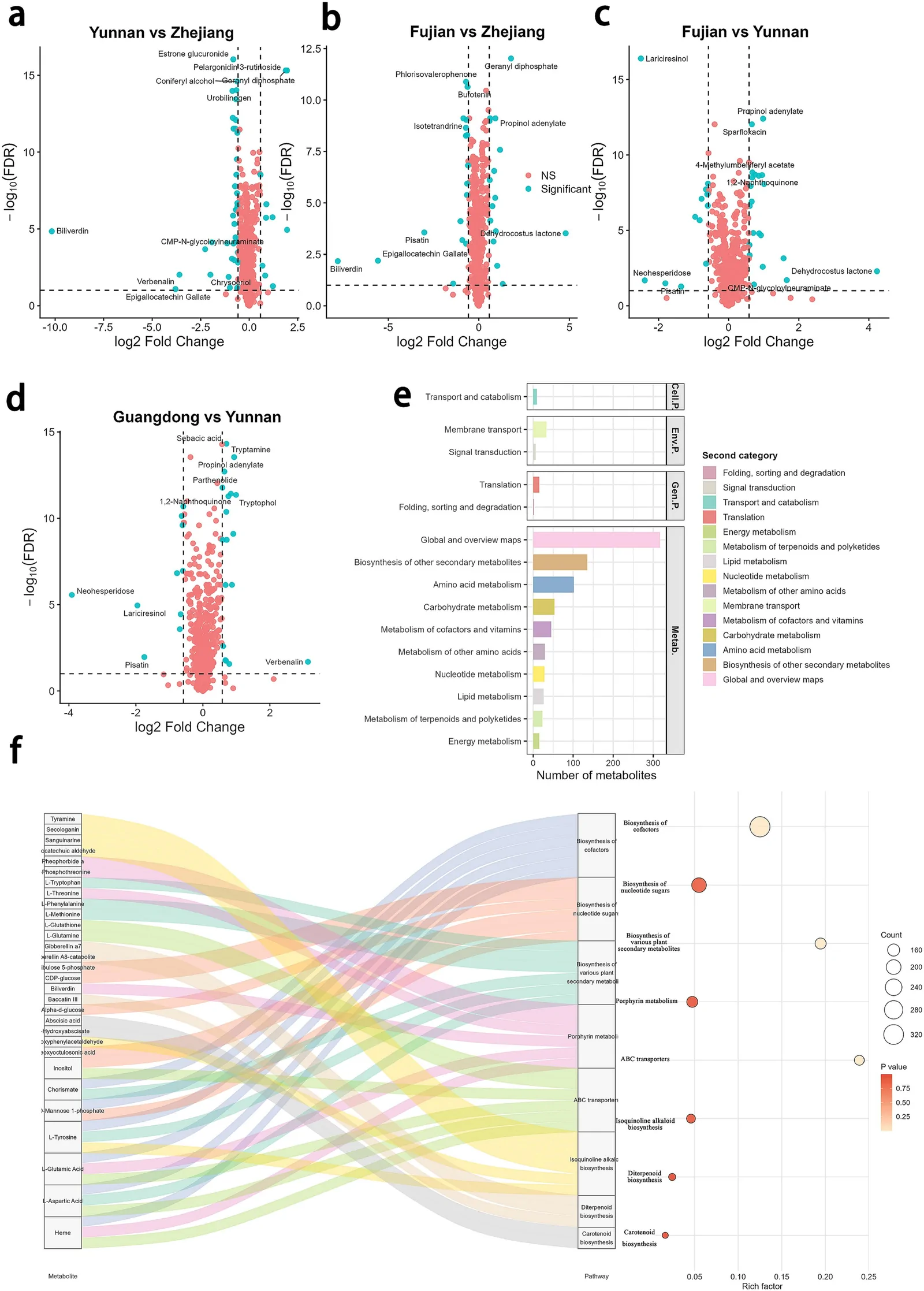
Differential screening and pathway-level characterization of the non-volatile metabolites in candied *Prunus mume* (CPM) across origins. (a–d) Volcano plots of differential non-volatile metabolites in representative inter-provincial comparisons. For these comparisons, samples from all origins within each province were grouped by province and analyzed jointly. Non-volatile metabolites with a FDR < 0.05 and a fold change (FC) ≥ 1.5 or ≤ 0.67 were considered differential. Dashed lines indicate the corresponding FDR and FC thresholds. (e) KEGG functional classification of annotated non-volatile metabolites. (f) Sankey diagram linking representative discriminatory non-volatile metabolites to KEGG pathways, with pathway enrichment shown on the right. In the pathway enrichment plot, bubble size represents the number of mapped metabolites, and color intensity represents the P value of pathway enrichment. Each origin included three independent biological replicates (*n* = 3).

Although multivariate analysis showed overall separation among origins, the extent of pairwise inter-provincial differentiation varied. For the volcano-plot analysis, samples from all origins within each province were grouped and analyzed jointly in pairwise province-level comparisons using the criteria described in Section 2.6. Representative comparisons are shown in Fig. 6a–6d, while the remaining comparisons are presented in **Fig. S2a–2** **f**. Notably, several metabolites, such as biliverdin, lariciresinol, neohesperidose, dehydrocostus lactone, pisatin, and verbenalin, recurred across multiple volcano plots (Fig. 6a–6d). The repeated occurrence of these metabolites across several comparisons suggests that they contributed to inter-origin differentiation mainly through differences in relative abundance, rather than acting as metabolites unique to a particular origin. Together with the largely shared metabolite repertoire across origins, these recurrent differential features indicate that origin-dependent differentiation in CPM was mainly reflected by distinct abundance-change patterns across province-pair comparisons, especially involving flavonoid glycosides, pigment-related metabolites, lignans, and glycoside-type compounds. This metabolite pool was therefore further refined using RF ranking and RF-derived SHAP interpretation to identify the non-volatile metabolites contributing most strongly to RF model output across origins (Liu et al., 2025).

#### 3.3.2 Candidate non-volatile metabolites prioritized by RF and SHAP

**Fig. 7:**
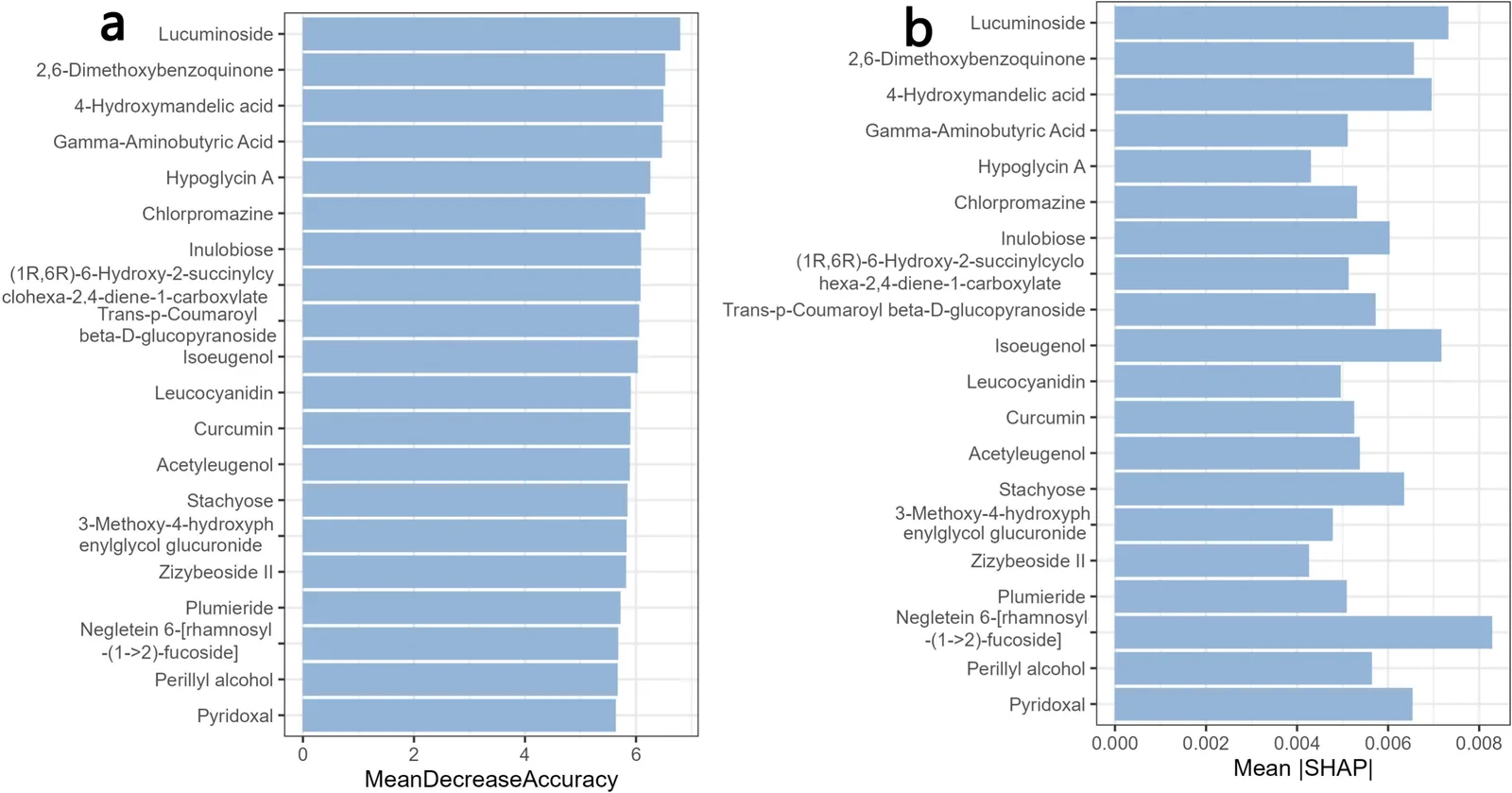
Prioritization of key non-volatile metabolites associated with origin-related metabolomic differentiation in candied *Prunus mume* (CPM) using random forest (RF) and Shapley additive explanations (SHAP) analyses. (a) Top 20 non-volatile metabolites ranked by RF importance based on MeanDecreaseAccuracy. (b) SHAP values of the same RF-selected top 20 metabolites, expressed as mean absolute SHAP values (Mean |SHAP|). Each origin included three independent biological replicates (*n* = 3).

To further prioritize the metabolites contributing most strongly to origin-dependent differentiation, Random Forest (RF) was used to rank metabolites according to their importance for distinguishing origins, whereas Shapley additive explanations (SHAP) were subsequently used to interpret how the RF-selected metabolites contributed to model predictions (Xue et al., 2026). RF importance highlighted a compact panel of candidate differentiating metabolites, with representative top-ranked features including Lucuminoside, 2,6-dimethoxybenzoquinone, 4-hydroxymandelic acid, gamma-aminobutyric acid, Inulobiose, trans-p-coumaroyl beta-D-glucopyranoside, Isoeugenol, Leucocyanidin, Curcumin, Stachyose, Perillyl alcohol, and Pyridoxal (Fig. 7a). These metabolites covered diverse chemical categories, including bioactive functional metabolites such as glycosides and phenolic derivatives, amino-acid-related metabolites, carbohydrates, and phenylpropanoid-related compounds, indicating that origin-related metabolomic differentiation was associated with coordinated changes across multiple metabolite groups rather than with a single chemical class. RF-derived SHAP analysis further quantified how the same RF-selected top 20 metabolites contributed to RF model output across origins (Fig. 7b). Among these RF-prioritized features, Lucuminoside, Isoeugenol, 4-hydroxymandelic acid, 2,6-dimethoxybenzoquinone, Pyridoxal, Stachyose, and Inulobiose showed relatively high mean absolute SHAP values, suggesting that these metabolites made stronger contributions to the overall RF classification output.

**Fig. 8:**
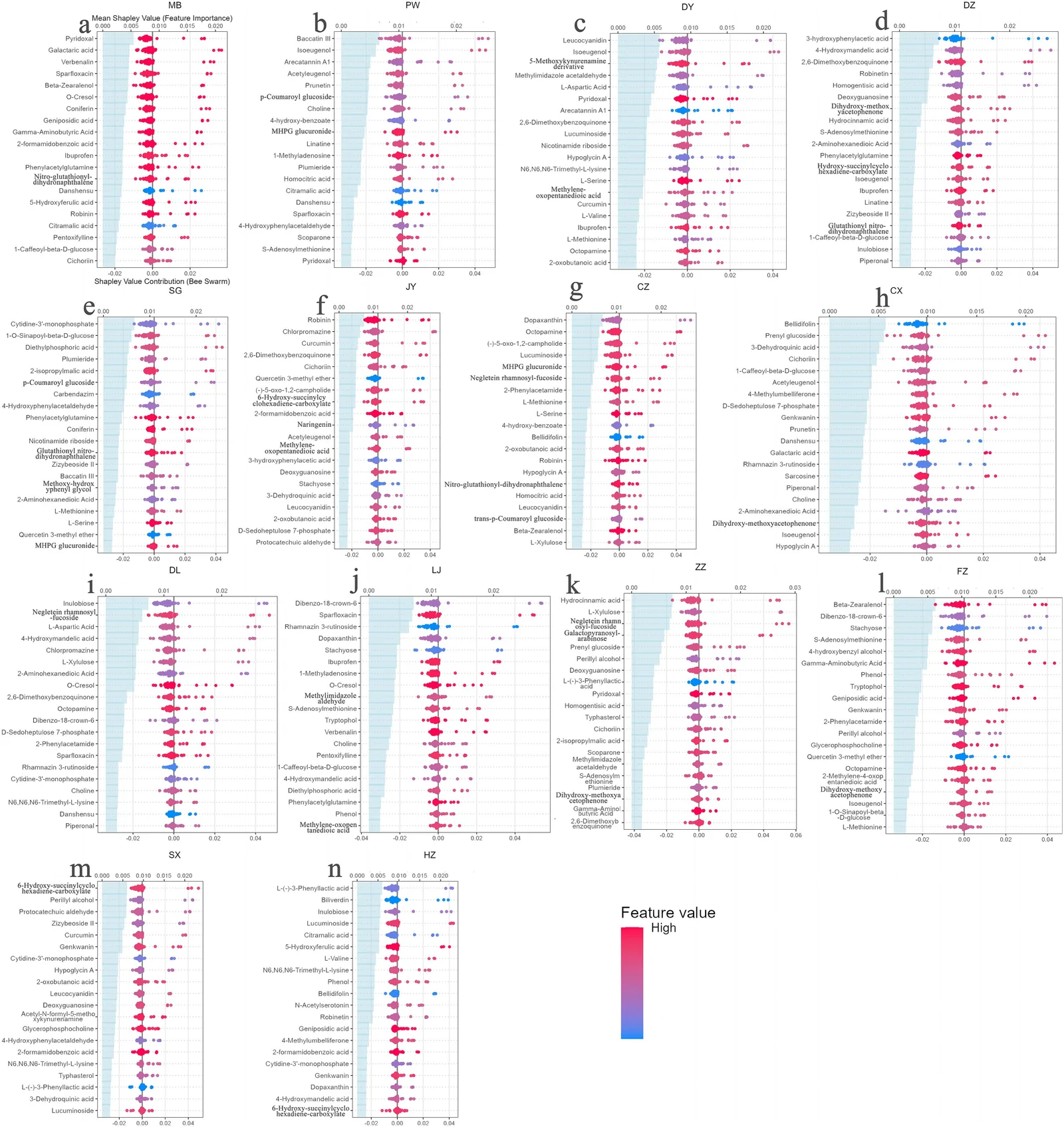
Class-specific SHAP summaries of key non-volatile metabolites across origins of CPM. Bars indicate feature importance, and points indicate SHAP contributions. Colors represent relative feature abundance from low (blue) to high (red). Positive and negative SHAP values indicate positive and negative contributions, respectively, to the prediction of the corresponding origin. Each origin included three independent biological replicates (*n* = 3). (For interpretation of the references to color in this figure legend, the reader is referred to the web version of this article.)

Origin-specific SHAP summaries were further generated from the same multiclass RF model to examine class-dependent metabolite contributions across origins (Fig. 8). These summaries indicated that different origins were not characterized by a uniform marker set. Several RF-prioritized metabolites, including Negletein 6-[rhamnosyl-(1 → 2)-fucoside], Lucuminoside, Perillyl alcohol, 2,6-dimethoxybenzoquinone, Pyridoxal, Isoeugenol, Stachyose, Curcumin, and Inulobiose, contributed differently across origins, indicating that their relative importance was origin dependent rather than uniform across all classes. Overall, these origin-specific SHAP patterns indicate that the same group of candidate differentiating metabolites contributed differently to the classification of different origins, thereby providing a more detailed interpretation of origin-related metabolomic differentiation.

#### 3.3.3 Pathway-level interpretation of discriminative non-volatile metabolites

Because the chemical-class overview had already shown broad metabolite coverage in CPM (Fig. 5d), KEGG classification was used to further examine which metabolic systems were mainly represented by these annotated metabolites. Based on the Kyoto Encyclopedia of Genes and Genomes (KEGG) pathway annotation framework (Kanehisa et al., 2022), annotated metabolites were assigned mainly to global and overview maps, followed by biosynthesis of other secondary metabolites, amino acid metabolism, carbohydrate metabolism, metabolism of cofactors and vitamins, and nucleotide metabolism (Fig. 6e). This distribution indicates that the non-volatile metabolite profile of CPM was not dominated by a single pathway category, but involved both primary metabolism-related pathways, such as amino acid and carbohydrate metabolism, and specialized metabolism-related pathways, such as secondary metabolite biosynthesis. Such a pathway-level distribution is consistent with the broad chemical diversity observed in the metabolite profile and suggests that origin-related differentiation may reflect coordinated variation in taste-related amino acids and carbohydrates, cofactor/vitamin-associated metabolites, and bioactive functional metabolites (Jiang et al., 2021; Zhao et al., 2024).

Metabolite-to-pathway mapping and KEGG pathway enrichment were then used to place discriminative metabolites into specific pathway contexts. Representative pathways included biosynthesis of cofactors, biosynthesis of nucleotide sugars, biosynthesis of various plant secondary metabolites, porphyrin metabolism, ABC transporters, isoquinoline alkaloid biosynthesis, diterpenoid biosynthesis, and carotenoid biosynthesis (Fig. 6f). These pathways correspond to cofactor metabolism, sugar-nucleotide conversion, secondary metabolite accumulation, pigment-related metabolism, and membrane transport processes, suggesting that origin-related differences involved coordinated variation across primary metabolism, secondary metabolism, and transport-related processes. Together, these KEGG classification and pathway-enrichment results provide pathway-level biochemical context for the origin-dependent metabolomic differentiation observed in the multivariate and RF–SHAP analyses.

### 3.4 Integrated analysis of VOCs, metabolites, and quality traits

**Fig. 9:**
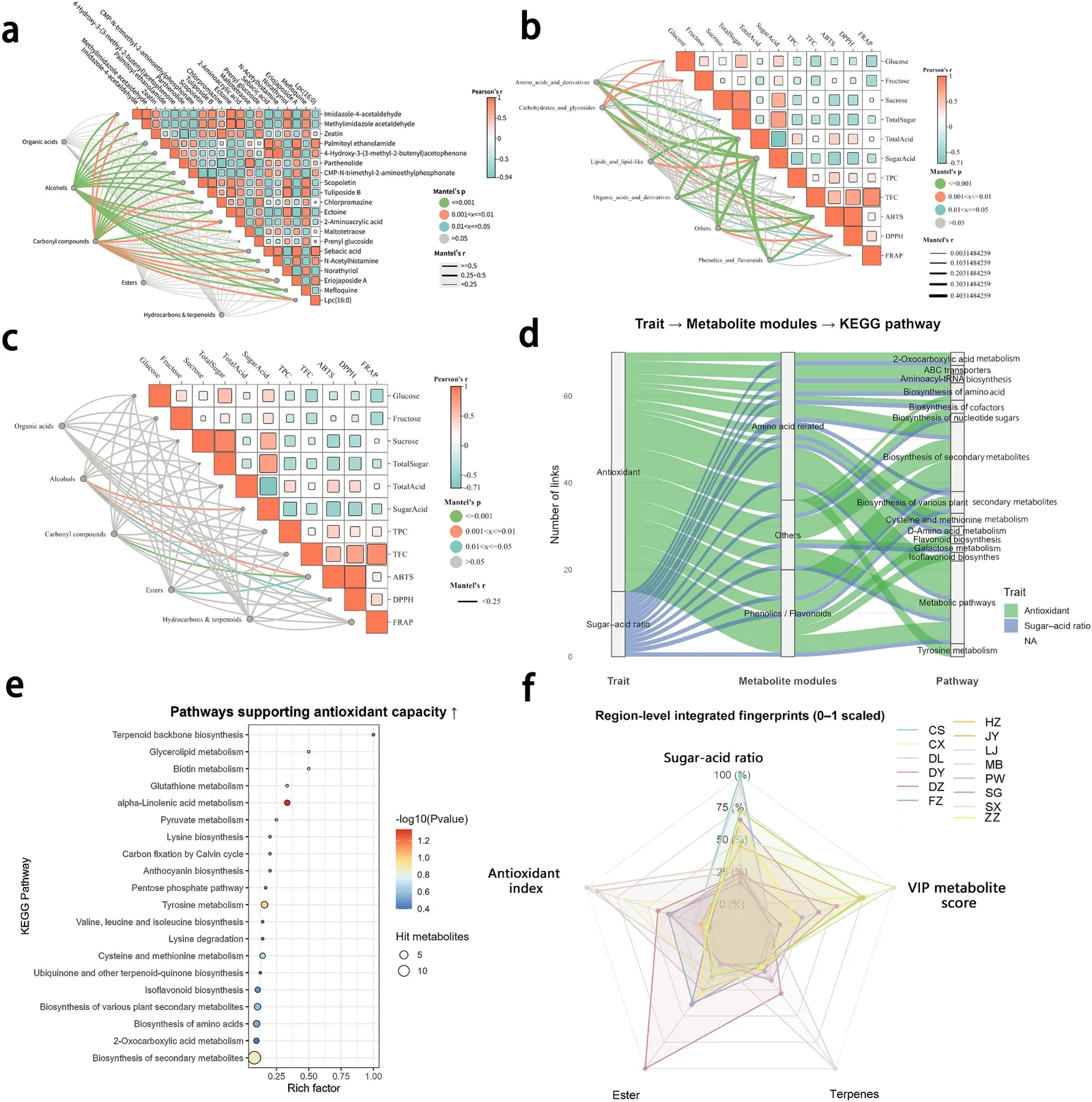
Integrated analysis of VOC, non-volatile metabolite, and trait associations. (a) Correlation network between major VOC classes and representative differential metabolites. (b) Mantel test-based correlation network between metabolite classes and physicochemical/functional traits. (c) Mantel test-based correlation network between VOC classes and physicochemical/functional traits. (d) Sankey diagram linking antioxidant-related traits and sugar–acid ratio to metabolite modules and enriched pathways. (e) KEGG enrichment of antioxidant-associated metabolites. (f) Origin-level integrated fingerprints of CPM from 14 origins. The five evaluation dimensions were sugar–acid ratio, antioxidant index, ester score, terpene score, and variable importance in projection (VIP) metabolite score. For each origin, the mean value of three independent biological replicates (*n* = 3) was calculated. The sugar–acid ratio, each antioxidant-related indicator, the total relative abundances of ester and terpene VOCs, and the abundance of each selected non-volatile metabolite were normalized across the 14 origins using min–max scaling to a range of 0–100: $X_{\text{score}}=\frac{X-X_{\min}}{X_{\max}-X_{\min}}\times 100$, where X represents the mean value of a given indicator for one origin, and $X_{\min}$ and $X_{\max}$ represent the minimum and maximum values of that indicator across the 14 origins, respectively. The antioxidant index was calculated as the mean of the individually normalized TPC, TFC, DPPH radical-scavenging activity, ABTS radical-scavenging activity, and RP values. The ester and terpene scores were obtained by min–max normalizing the total relative abundances of VOCs assigned to the corresponding chemical classes across the 14 origins. The top 30 non-volatile metabolites with the highest VIP values were selected, and the VIP metabolite score was calculated as the mean of their individually normalized abundances. Higher scores indicate relatively higher levels of the corresponding dimension among the 14 origins. Mantel tests were performed using correlation-based distance matrices, and significance was assessed by permutation testing (*P* < 0.05). Pearson correlation coefficients are shown in the correlation networks.

Integrative correlation and network analyses were performed to link VOCs, non-volatile metabolites, and quality-related traits across the fourteen origins (Fig. 9a–9f). For integrative analysis, aldehydes and ketones were merged into a single carbonyl-compound class. The VOC–metabolite association network revealed structured relationships between major VOC classes and representative differential metabolites, with alcohols and carbonyl compounds displaying relatively extensive Mantel-supported connections (Fig. 9a). These results indicate that origin-dependent aroma variation was associated with the broader non-volatile metabolic background of CPM rather than being determined by isolated VOC_S_ alone.

Mantel-based integrative analysis further supported the coupling between chemical composition and quality-related traits. At the VOC-class level, associations with physicochemical and functional traits were selective rather than uniform, indicating that only some aroma categories were significantly linked to sugar/organic-acid-related and antioxidant-related parameters (Fig. 9c). In contrast, metabolite–trait correlations showed a clearer functional pattern (Fig. 9b): phenolic/flavonoid-related metabolites were mainly associated with antioxidant-related indices, whereas carbohydrate/glycoside-related and organic acid-related metabolites were more closely linked to sugar- and acidity-related variables. Representative discriminative metabolites from the VIP, volcano-plot, and RF-SHAP analyses were consistent with these class-level patterns. These included bioactive functional metabolites such as flavonoid glycosides (Quercetin 3-O-glucosyl-xyloside and 6-Hydroxykaempferol 3-glucoside) and phenylpropanoid/phenolic-related compounds (Isoeugenol), as well as Lucuminoside and carbohydrate/glycoside-related metabolites such as Stachyose, Inulobiose, and neohesperidose. Together, these results indicate two major trait-related dimensions underlying origin-dependent quality differentiation in CPM: an antioxidant-related dimension and a sugar–acid-related dimension (Fig. 9b-9d). These findings are in line with recent postharvest fruit profiling studies showing that organic acids, flavonoids, sugar–acid balance, and VOC profiles are closely associated with fruit quality and nutritional value (Garcia et al., 2025).

Pathway-level analysis further showed that these trait-associated differences involved glutathione metabolism, the pentose phosphate pathway, alpha-linolenic acid metabolism, cysteine and methionine metabolism, tyrosine metabolism, amino acid biosynthesis, and secondary metabolite biosynthesis (Fig. 9d, e). These pathways provided pathway-level biochemical context for the two quality-related dimensions. Specifically, antioxidant-related traits were mainly linked to phenolic/flavonoid and redox-related metabolism, whereas sugar-acid-related variation was more closely associated with carbohydrate- and organic-acid-related metabolic modules (Stincone et al., 2015; Wang et al., 2024). Therefore, the integrated analysis shifted the interpretation from compound-level differentiation to quality-related metabolic organization. The network results further linked ROAV-based aroma profiles, non-volatile metabolic background, and quality traits into interpretable antioxidant-related and sugar–acid-related patterns under the unified processing regime (Zhao et al., 2022).

Across the five major producing regions, CPM exhibited layered regional differentiation in VOC organization, non-volatile metabolite abundance patterns, and quality-related traits. Sichuan showed pronounced within-region heterogeneity, with MB, PW, DY, and DZ displaying markedly different VOC and VIP-ranked metabolite profiles rather than a single province-wide chemical pattern. Their VOC characteristics ranged from terpene- and alkane-related profiles to aldehyde- and organic-acid-related profiles, while MB additionally showed a sweet-oriented quality tendency. Guangdong was characterized by an alcohol-rich VOC background with additional ester and aldehyde differentiation. Province-level metabolome comparisons showed relatively higher verbenalin abundance in Guangdong than in Yunnan, whereas lariciresinol, neohesperidose, and pisatin were relatively higher in Yunnan. CZ was further distinguished by its high sugar–acid ratio. Fujian also showed an alcohol-rich VOC background with secondary ester- and terpenoid-related contributions. Its non-volatile metabolite profile showed relatively higher dehydrocostus lactone and propinol adenylate abundances than Yunnan or Zhejiang, whereas lariciresinol, neohesperidose, and pisatin were relatively higher in Yunnan. Yunnan showed alcohol–aldehyde co-dominance together with relatively prominent lignan-, glycoside-, and flavonoid-related metabolic features, including lariciresinol, neohesperidose, pisatin, and pelargonidin 3-rutinoside in the relevant province-level comparisons. This pattern was accompanied by the acid-oriented profile of CX and the stronger phenolic, flavonoid, and antioxidant-related characteristics of DL and LJ. Zhejiang likewise showed alcohol–aldehyde co-dominance but differed from Yunnan in the relative contributions of individual odor-active compounds. Its non-volatile metabolite profile showed relatively higher biliverdin, verbenalin, and epigallocatechin gallate abundances than Yunnan, together with pigment-related metabolites such as Hv-NCC-1. Overall, the five regions shared a broadly similar non-volatile metabolite repertoire, whereas regional differentiation was mainly expressed through different relative abundance patterns of recurrent metabolites, region-dependent VOC organization, and contrasting sugar–acid and antioxidant traits. Based on this regional synthesis, the fourteen origins were further compared using integrated fingerprints that were min–max scaled from 0 to 100 across five dimensions: sugar–acid ratio, antioxidant index, ester score, terpene score, and VIP metabolite score (Fig. 9f).

Origin differences were mainly reflected in distinct directional patterns rather than uniform shifts across all dimensions. These integrated fingerprints were therefore used to describe origin-level quality tendencies rather than to establish strict groupings or categorical classifications. CX showed an acid-oriented tendency, reflected by its low sugar–acid-ratio score. DL and LJ were associated with stronger antioxidant-related features. MB exhibited a relatively high terpene score, and CZ showed the highest sugar–acid-ratio score. PW, HZ, and SX showed relatively balanced multidimensional profiles without clear dominance of a single dimension, whereas the remaining origins showed different intermediate combinations of the five indicators. Collectively, the integration of sugar–acid balance, antioxidant-related functional traits, ester- and terpene-related aroma profiles, and VIP-ranked non-volatile metabolite patterns provided a multidimensional description of origin-level quality differences (Li et al., 2022).

## 4 Conclusion

This study characterized origin-related differences in the quality chemistry of candied *Prunus mume* prepared under a unified candying regime. Fruits from fourteen geographical origins were collected during the same harvesting season and subjected to an identical salt-curing and sugar-preservation procedure, providing a consistent experimental basis for origin-level comparison. The integration of quality-trait assays, HS-SPME-GC–MS VOC profiling, relative odor activity value analysis, weighted correlation network analysis, non-volatile metabolite profiling, RF–SHAP interpretation, and cross-layer correlation analysis provided an integrated analytical framework for interpreting quality differentiation across physicochemical, volatile, non-volatile, and functional dimensions. During candying, the osmotic gradient generated by the external sugar solution likely promoted water efflux and sugar uptake, thereby modifying the concentrations and relative balance of soluble sugars, organic acids, glycosides, and other water-soluble metabolites. Meanwhile, phenolic and flavonoid compounds may have undergone oxidation and related transformations during salt curing, air drying, and sugar preservation, affecting their abundance and antioxidant-related properties. Origin-dependent differences in carbohydrate-, organic-acid-, phenolic-, flavonoid-, amino-acid-, fatty-acid-, phenylpropanoid-, and terpenoid-related metabolic backgrounds may therefore have influenced the responses of the raw materials to the same candying conditions. These processes provide a plausible biochemical link between geographical origin, metabolite composition, sugar–acid balance, aroma formation, and antioxidant-related function. The integrated results indicated that antioxidant-related traits were mainly associated with phenolic, flavonoid, and redox-related metabolism, whereas sugar–acid-related variation was more closely linked to carbohydrates, glycosides, and organic acids. Aroma differentiation reflected the combined contributions of shared key odorants, origin-associated odor-active compounds, coordinated VOC organization, and differences in volatile precursor backgrounds. RF–SHAP further prioritized candidate non-volatile metabolites associated with origin differentiation, while cross-layer integration connected these metabolic patterns with flavor- and function-related traits. Overall, this study provided an integrated perspective on the relationships among geographical origin, metabolites, flavor, and functional traits in CPM and illustrated the potential utility of combining a unified processing design with multi-omics and interpretable machine-learning approaches. The resulting multidimensional chemical profiles may provide useful chemical information for origin-oriented raw-material selection, quality grading, differentiated product development, and future geographical indication-related quality evaluation. The phenolic- and flavonoid-associated antioxidant characteristics also suggest potential nutritional value, although the present in vitro assays do not directly demonstrate physiological health effects. These fingerprints may support regional product positioning and consumer recognition, but their relationships with sensory preference and market acceptance require further validation. Future studies using paired fresh and candied fruits and time-resolved sampling during processing are needed to directly evaluate sugar osmosis, polyphenol oxidation, and volatile-precursor conversion. Targeted validation of candidate compounds, together with analyses of color, texture, nutritional bioavailability, sensory quality, and consumer acceptance, will further clarify how these chemical patterns translate into perceived product quality and industrial value.

## CRediT authorship contribution statement

**Jia-li Liao:** Writing – review & editing, Writing – original draft, Methodology, Investigation, Data curation. **Yi-xiao Xiao:** Writing – review & editing, Writing – original draft, Methodology, Investigation, Formal analysis. **Jia-xin Zhong:** Writing – review & editing, Investigation, Formal analysis. **Yi-jing He:** Writing – review & editing, Validation, Formal analysis. **Wen-zhang Qian:** Writing – review & editing, Investigation, Formal analysis. **Ji-cheng Chen:** Writing – review & editing, Supervision. **Jin-lin Guo:** Writing – review & editing, Supervision. **Shun Gao:** Writing – review & editing, Supervision, Project administration, Funding acquisition, Conceptualization.

## Ethics declaration

The author(s) declare(s) that the study does not involve humans nor animal subjects.

## Declaration of competing interest

The authors declare that they have no known competing financial interests or personal relationships that could have appeared to influence the work reported in this paper.

## Data Availability

Data will be made available on request.
