# Supplementary material for: Origin-dependent chemical profiles and quality differentiation of candied Prunus mume: Insights from metabolomics, GC–MS volatile profiling, and interpretable machine learning
Source: Food Chem X. 2026 Sep 1;39:104391. doi: 10.1016/j.fochx.2026.104391 (PMC13571749; doi:10.1016/j.fochx.2026.104391)
Supplement: Supplementary file 1 [file mmc1.docx]

**Supplementary materials: Figures S1-S3 and Tables S1-S5**

**Fig. S1**. Volcano plots of differential volatile organic compounds (VOCs) in candied *Prunus mume* (CPM) from representative province-level comparisons. The x-axis shows log₂ fold change (FC), and the y-axis shows −log_10_ (false discovery rate (FDR)). Dashed lines indicate the FC and FDR thresholds. Blue dots indicate significant VOCs, and red dots indicate non-significant VOCs. Each origin included three independent biological replicates (n = 3).

**Fig. S2.** Volcano plots of differential non-volatile metabolites in candied *Prunus mume* (CPM) from representative province-level comparisons. For each comparison, samples from all origins within each province were grouped by province and analyzed jointly. The x-axis shows log₂ fold change (FC), and the y-axis shows −log₁₀ false discovery rate (FDR). Non-volatile metabolites with FDR < 0.05 and FC ≥ 1.5 or ≤ 0.67 were considered differential. Dashed lines indicate the corresponding FC and FDR thresholds. Blue dots indicate differential metabolites, and red dots indicate non-differential metabolites. Each origin included three independent biological replicates (n = 3).

**Fig. S3**. Hierarchical clustering heatmap of volatile organic compounds (VOCs) in candied *Prunus mume* (CPM) from 14 geographical origins. For each origin, the mean of three independent biological replicates was used (n = 3). Relative abundances were standardized row-wise using Z-scores. Red and blue indicate higher and lower standardized relative abundances, respectively.

**Supplementary Table S1.** Geographical, climatic, and phenological information of *Prunus mume* from fourteen geographical origins

| **Code** | **Production origins** | **Coordinates** | **Mean temperature during May–June 2025 (°C)** | **Cumulative precipitation during May–June 2025 (mm)** | **flowering period** | **harvest/maturity period** | **Fruit developmental period (d)** |
| --- | --- | --- | --- | --- | --- | --- | --- |
| MB | Mabian County, Leshan City, Sichuan, China | 28.425–29.071° N, 103.244–103.828° E | 20.26 | 314.37 | Late Feb | Late Jun | ~120 |
| PW | Pingwu County, Mianyang City, Sichuan, China | 31.992–33.045° N, 103.842–104.987° E | 15.27 | 330.10 | Late Feb | Late Jun | ~120–140 |
| DY | Dayi County, Chengdu City, Sichuan, China | 30.417–30.817° N, 102.983–103.750° E | 21.77 | 387.10 | Late Feb | Early Jun | ~100 |
| DZ | Dachuan District, Dazhou City, Sichuan, China | 30.817–31.550° N, 106.983–107.833° E | 23.81 | 261.93 | Late Feb | Late May | ~90–100 |
| CZ | Raoping County, Chaozhou City, Guangdong, China | 23.467–24.233° N, 116.583–117.183° E | 25.67 | 438.24 | Mid-Jan | Early May | ~100-110 |
| JY | Puning City, Jieyang City, Guangdong, China | 23.094–23.530° N, 115.719–116.351° E | 25.55 | 409.74 | Mid-Jan | Early May | ~100-110 |
| SG | Wengyuan County, Shaoguan City, Guangdong, China | 24.125–24.621° N, 113.301–114.301° E | 24.17 | 561.39 | Mid-Jan | Early May | ~100–110 |
| CX | Chuxiong City, Chuxiong Prefecture, Yunnan, China | 24.500–25.250° N, 100.583–101.800° E | 21.77 | 387.10 | Mid-Jan | Mid-Jun | ~150 |
| DL | Weishan County, Dali Prefecture, Yunnan, China | 24.933–25.533° N, 99.917–100.417° E | 17.50 | 409.33 | Mid-Jan | Late Jun | ~165–175 |
| LJ | Yulong County, Lijiang City, Yunnan, China | 25.983–27.933° N, 99.383–101.517° E | 16.10 | 512.03 | Mid-Jan | Late May | ~125–135 |
| ZZ | Zhao’an County, Zhangzhou City, Fujian, China | 23.583–24.183° N, 116.917–117.367° E | 24.52 | 447.23 | Early Jan | Early May | ~115–120 |
| FZ | Yongtai County, Fuzhou City, Fujian, China | 25.650–26.083° N, 118.383–119.200° E | 22.97 | 359.87 | Mid-Jan | Early May | ~110 |
| SX | Shengzhou City, Shaoxing City, Zhejiang, China | 29.329–29.832° N, 120.456–121.115° E | 23.78 | 578.48 | Early–mid Mar | Early May | ~50–65 |
| HZ | Jiande City, Hangzhou City, Zhejiang, China | 29.217–29.767° N, 118.900–119.750° E | 23.66 | 537.63 | Early–mid Mar | Early May | ~50–65 |

**Notes:** Mean temperature and cumulative precipitation refer to the period from May to June 2025 and were obtained from the ERA5-Land monthly averaged dataset provided through the Copernicus Climate Data Store (CDS; https://cds.climate.copernicus.eu/) based on the geographical coordinates of the corresponding sampling origins. Flowering and harvest/maturity periods represent the reference phenological information of the corresponding production regions. Fruit developmental period was defined as the interval from full bloom to commercial harvest or maturity. “~” indicates an approximate value.

**Table S2.** Peel and flesh color parameters and fruit firmness of fresh *Prunus mume* fruits from fourteen geographical origins before processing.

| **Origin** | **Peel color** | | | **Flesh color** | | | **Firmness (kg/cm²)** |
| --- | --- | --- | --- | --- | --- | --- | --- |
|  | ***L**** | ***a**** | ***b**** | ***L**** | ***a**** | ***b**** |  |
| MB | 38.79 ± 3.25 | −2.34 ± 0.96 | 21.22 ± 1.52 | 34.01 ± 2.61 | −0.98 ± 0.60 | 18.61 ± 0.72 | 19.53 ± 2.02 |
| PW | 36.51 ± 2.51 | −2.45 ± 0.70 | 20.70 ± 1.43 | 34.66 ± 3.32 | −0.39 ± 0.42 | 19.31 ± 1.86 | 18.70 ± 2.44 |
| DY | 35.98 ± 3.20 | −2.22 ± 0.61 | 20.82 ± 1.87 | 36.80 ± 1.06 | 0.50 ± 0.90 | 19.12 ± 0.58 | 15.83 ± 0.96 |
| DZ | 35.19 ± 2.93 | −1.69 ± 1.07 | 21.85 ± 2.36 | 33.02 ± 2.33 | −0.92 ± 1.43 | 18.59 ± 1.47 | 17.90 ± 2.88 |
| SG | 36.17 ± 2.60 | −2.05 ± 2.14 | 21.28 ± 0.95 | 38.93 ± 0.70 | −1.13 ± 0.62 | 19.56 ± 0.89 | 18.43 ± 2.11 |
| JY | 35.62 ± 2.95 | −2.36 ± 1.13 | 21.04 ± 2.08 | 39.35 ± 1.73 | −1.16 ± 0.16 | 21.04 ± 0.71 | 16.60 ± 0.95 |
| CZ | 35.61 ± 4.43 | −2.09 ± 0.99 | 21.86 ± 2.30 | 35.85 ± 1.82 | 0.06 ± 0.65 | 21.62 ± 2.46 | 18.67 ± 2.05 |
| CX | 38.26 ± 2.96 | −2.39 ± 1.30 | 21.49 ± 2.43 | 38.14 ± 0.84 | −0.29 ± 0.20 | 22.66 ± 1.08 | 18.80 ± 1.21 |
| DL | 37.61 ± 2.70 | −2.20 ± 1.28 | 22.95 ± 1.43 | 38.45 ± 2.96 | −0.41 ± 0.58 | 21.45 ± 2.26 | 18.43 ± 2.33 |
| LJ | 38.42 ± 2.91 | −2.17 ± 1.86 | 23.49 ± 2.41 | 31.79 ± 1.68 | −1.92 ± 0.12 | 19.61 ± 0.96 | 17.63 ± 0.98 |
| ZZ | 36.56 ± 2.92 | −2.73 ± 1.10 | 21.36 ± 2.18 | 35.24 ± 1.46 | −1.21 ± 0.38 | 20.74 ± 1.41 | 18.40 ± 1.21 |
| FZ | 35.89 ± 2.85 | −2.19 ± 0.95 | 19.70 ± 2.47 | 35.91 ± 1.62 | −0.54 ± 0.35 | 21.02 ± 1.29 | 19.00 ± 1.25 |
| SX | 35.30 ± 3.89 | −1.91 ± 0.99 | 21.46 ± 2.15 | 37.78 ± 1.42 | −1.45 ± 0.44 | 19.69 ± 1.05 | 18.73 ± 0.78 |
| HZ | 36.13 ± 3.02 | −1.89 ± 0.94 | 20.38 ± 2.28 | 36.31 ± 1.79 | −1.67 ± 0.65 | 19.59 ± 1.97 | 19.40 ± 1.20 |

Notes: Values are expressed as mean ± standard deviation (SD). Peel color and fruit firmness were measured on ten individual fruits per origin (n = 10). Flesh color values were calculated from the three measurements provided for each origin (n = 10). Color was expressed using the CIELAB system, where *L** represents lightness, *a** represents the green-to-red coordinate, and *b** represents the blue-to-yellow coordinate. Fruit firmness was measured using a GY-3 fruit firmness tester equipped with a Φ8 mm probe and expressed as kg/cm².

**Supplementary Table S3.** Relative contents of identified volatile organic compounds (VOCs) in candied *Prunus mume* (CPM) from 14 origins.

| **Name** | **CAS** | **MB** | **PW** | **DY** | **DZ** | **CZ** | **JY** | **SG** | **CX** | **DL** | **LJ** | **ZZ** | **FZ** | **SX** | **HZ** |
| --- | --- | --- | --- | --- | --- | --- | --- | --- | --- | --- | --- | --- | --- | --- | --- |
| 2-Ethyl-1-hexanol | 104 - 76 - 7 | - | - | - | - | 0.04 ± 0.01 | 0.07 ± 0.02 | 0.02 ± 0.01 | 0.07 ± 0.02 | 0.06 ± 0.02 | 0.11 ± 0.02 | 0.09 ± 0.05 | 0.11 ± 0.03 | 0.05 ± 0.01 | 0.06 ± 0.01 |
| 1-Nonanol | 143 - 08 - 8 | - | - | - | - | 0.09 ± 0.03 | - | 0.04 ± 0.01 | - | - | 0.16 ± 0.02 | 0.14 ± 0.05 | 0.06 ± 0.03 | - | - |
| 1-Octanol | 111 - 87 - 5 | - | - | - | - | 0.12 ± 0.01 | 0.36 ± 0.06 | 0.05 ± 0.01 | 0.09 ± 0.03 | 0.09 ± 0.04 | - | 0.25 ± 0.09 | 0.14 ± 0.05 | 0.06 ± 0.02 | 0.16 ± 0.02 |
| 1-Octen-3-ol | 3391 - 86 - 4 | - | - | - | - | 0.20 ± 0.00 | 0.15 ± 0.03 | - | 0.10 ± 0.03 | - | - | - | - | - | - |
| (Z)-2-Octen-1-ol | 26001 - 58 - 1 | - | - | - | - | 0.08 ± 0.01 | - | 0.02 ± 0.01 | 0.06 ± 0.02 | 0.05 ± 0.02 | 0.14 ± 0.06 | - | - | - | - |
| Benzyl alcohol | 100 - 51 - 6 | - | - | 0.23 ± 0.05 | 0.35 ± 0.13 | 1.00 ± 0.02 | 0.27 ± 0.02 | 0.05 ± 0.01 | 7.18 ± 1.77 | 9.97 ± 4.62 | 1.70 ± 0.48 | 0.34 ± 0.12 | 0.36 ± 0.12 | 0.94 ± 0.28 | 4.77 ± 0.41 |
| Phenylethyl alcohol | 60-12-8 | - | - | - | - | 0.62 ± 0.08 | 2.83 ± 0.59 | 0.28 ± 0.02 | 0.67 ± 0.16 | 1.25 ± 0.56 | 3.54 ± 1.18 | 2.72 ± 0.06 | 0.50 ± 0.04 | 0.65 ± 0.22 | 0.31 ± 0.04 |
| 3-Methyl-1-butanol | 123-51-3 | - | - | - | - | - | 5.93 ± 0.71 | 0.80 ± 0.18 | 2.57 ± 0.62 | 3.23 ± 1.48 | 7.61 ± 1.94 | 6.53 ± 1.08 | 1.06 ± 0.24 | 1.98 ± 0.61 | 0.72 ± 0.05 |
| 1-Heptanol | 111-70-6 | - | - | - | - | - | 0.19 ± 0.02 | 0.04 ± 0.00 | - | - | - | - | - | - | 0.15 ± 0.01 |
| 1-Hexanol | 111-27-3 | - | - | - | - | - | 0.80 ± 0.16 | 0.05 ± 0.02 | - | - | - | 0.81 ± 0.31 | 0.95 ± 0.20 | 0.22 ± 0.07 | - |
| 2-Methyl-1-propanol | 78-83-1 | - | - | - | - | - | 1.59 ± 0.36 | - | - | - | - | 1.46 ± 0.56 | - | - | - |
| 7-Octen-2-ol, 2-methyl-6-methylene- | 543-39-5 | 0.92 ± 0.20 | 0.06 ± 0.01 | - | - | - | 0.03 ± 0.01 | 0.02 ± 0.01 | - | - | - | 0.06 ± 0.03 | 0.10 ± 0.02 | - | - |
| 1,5,7-Octatrien-3-ol, 3,7-dimethyl- | 29957 - 43 - 5 | - | - | - | - | - | - | - | - | 0.52 ± 0.26 | 0.82 ± 0.29 | - | - | 0.22 ± 0.07 | 0.21 ± 0.02 |
| (E,E)-2,4-Heptadienal | 4313-03-5 | - | - | - | 0.54 ± 0.11 | 0.19 ± 0.03 | 0.24 ± 0.06 | - | 0.11 ± 0.02 | 0.26 ± 0.11 | 0.39 ± 0.07 | - | - | 0.17 ± 0.02 | 0.18 ± 0.06 |
| Benzaldehyde | 100 - 52 - 7 | - | - | 2.51 ± 0.24 | - | 1.90 ± 0.31 | - | - | 10.81 ± 8.65 | 14.61 ± 7.52 | - | - | - | 0.12 ± 0.04 | 5.06 ± 0.63 |
| 2,4-Dimethylbenzaldehyde | 15764 - 16 - 6 | 0.80 ± 0.13 | 0.19 ± 0.04 | - | 0.51 ± 0.23 | 0.09 ± 0.02 | 0.08 ± 0.04 | 0.06 ± 0.01 | 0.07 ± 0.03 | 0.09 ± 0.06 | 0.17 ± 0.04 | 0.17 ± 0.02 | 0.12 ± 0.04 | 0.07 ± 0.02 | 0.08 ± 0.02 |
| Nonanal | 124 - 19 - 6 | 0.96 ± 0.18 | 0.15 ± 0.03 | - | 0.62 ± 0.17 | 0.10 ± 0.01 | 0.13 ± 0.04 | - | - | - | - | - | - | - | - |
| (E)-2-Heptenal | 18829-55-5 | - | - | - | 1.01 ± 0.62 | - | 0.16 ± 0.07 | 0.04 ± 0.01 | 0.14 ± 0.06 | 0.14 ± 0.04 | 0.23 ± 0.06 | - | - | 0.24 ± 0.10 | 0.28 ± 0.11 |
| (E)-2-Nonenal | 18829-56-6 | - | - | - | - | - | - | - | - | - | - | - | - | 0.04 ± 0.02 | - |
| (E)-2-Octenal | 2548-87-0 | - | - | - | - | - | - | - | - | - | - | - | - | 0.28 ± 0.09 | - |
| Citral | 5392-40-5 | - | - | - | - | - | - | - | - | - | - | - | - | 0.04 ± 0.02 | - |
| Hexanal | 66-25-1 | - | - | - | - | - | - | - | - | - | - | - | - | 0.17 ± 0.05 | - |
| Octanal | 124-13-0 | - | - | - | 0.33 ± 0.01 | - | - | - | - | - | - | - | - | 0.05 ± 0.03 | - |
| Methyl benzoate | 93 - 58 - 3 | - | - | - | - | 0.04 ± 0.01 | 0.08 ± 0.02 | 0.02 ± 0.00 | - | - | - | 0.09 ± 0.06 | 0.11 ± 0.05 | - | 0.03 ± 0.01 |
| Ethyl benzoate | 93-89-0 | - | - | - | - | - | 0.17 ± 0.01 | - | 0.27 ± 0.09 | 0.28 ± 0.10 | - | - | - | 0.11 ± 0.04 | 0.07 ± 0.01 |
| Ethyl decanoate | 110-38-3 | - | - | - | - | - | 0.07 ± 0.02 | 0.04 ± 0.01 | 0.05 ± 0.02 | 0.16 ± 0.05 | - | 0.19 ± 0.04 | - | 0.04 ± 0.00 | - |
| Ethyl dodecanoate | 106-33-2 | - | - | - | - | - | 0.07 ± 0.05 | 0.02 ± 0.01 | 0.08 ± 0.02 | 0.07 ± 0.01 | - | 0.12 ± 0.05 | - | - | - |
| Nonyl acetate | 143 - 13 - 5 | - | - | - | - | - | - | 0.01 ± 0.00 | - | - | - | - | - | - | - |
| Ethyl acetate | 141 - 78 - 6 | - | - | - | - | - | - | 1.11 ± 0.24 | - | - | - | - | 0.72 ± 0.37 | - | - |
| Ethyl octanoate | 106 - 32 - 1 | - | - | - | 0.43 ± 0.18 | - | - | 0.04 ± 0.00 | - | - | - | 0.19 ± 0.04 | - | 0.09 ± 0.04 | - |
| 3-Hydroxy-2,2,4-trimethylpentyl isobutyrate | 77 - 68 - 9 | - | - | - | 0.29 ± 0.07 | - | - | 0.01 ± 0.00 | - | - | - | - | - | 0.02 ± 0.00 | - |
| 2-Phenylethyl acetate | 103-45-7 | - | - | - | - | - | - | - | 0.04 ± 0.00 | 0.08 ± 0.03 | 0.32 ± 0.05 | - | - | - | - |
| Benzyl acetate | 140-11-4 | - | - | - | - | - | - | - | 0.10 ± 0.03 | 0.21 ± 0.09 | - | - | - | - | 0.16 ± 0.02 |
| Ethyl hexadecanoate | 628 - 97 - 7 | - | - | - | - | - | - | - | 0.01 ± 0.01 | - | - | - | - | - | - |
| Methyl salicylate | 119 - 36 - 8 | - | - | - | - | - | - | - | 0.07 ± 0.02 | - | - | - | - | - | - |
| Ethyl hexanoate | 123-66-0 | - | - | - | - | - | - | - | - | - | - | 0.29 ± 0.29 | - | - | - |
| Hexyl acetate | 142 - 92 - 7 | - | - | - | - | - | - | - | - | - | - | - | 0.36 ± 0.18 | - | - |
| Butyl hexanoate | 626 - 82 - 4 | - | - | - | 2.00 ± 1.10 | - | - | - | - | - | - | - | - | - | - |
| Hexyl hexanoate | 6378 - 65 - 0 | - | - | - | 0.44 ± 0.06 | - | - | - | - | - | - | - | - | - | - |
| 2,2,5,5-Tetramethyltetrahydrofuran | 15045 - 43 - 9 | - | - | - | - | 0.18 ± 0.01 | - | - | - | - | - | - | - | - | - |
| 2(3H)-Furanone, 5-hexyldihydro- | 706-14-9 | - | 0.11 ± 0.04 | - | - | - | 0.18 ± 0.05 | 0.03 ± 0.00 | - | - | 0.15 ± 0.07 | - | 0.44 ± 0.10 | 0.07 ± 0.02 | - |
| 2-Butyltetrahydrofuran | 1004-29-1 | - | - | - | - | - | 0.03 ± 0.00 | - | - | - | - | - | - | - | - |
| 4-Methyldodecane | 6117 - 97 - 1 | - | 0.26 ± 0.11 | - | 0.38 ± 0.21 | 0.06 ± 0.01 | - | 0.03 ± 0.00 | - | - | - | - | - | - | - |
| Hexadecane | 544 - 76 - 3 | 2.19 ± 0.27 | 0.39 ± 0.29 | - | 0.64 ± 0.29 | 0.12 ± 0.06 | - | 0.07 ± 0.05 | 0.11 ± 0.08 | 0.08 ± 0.02 | 0.13 ± 0.06 | 0.20 ± 0.07 | 0.12 ± 0.06 | - | 0.11 ± 0.12 |
| Tetradecane | 629 - 59 - 4 | 1.59 ± 0.29 | - | - | 0.82 ± 0.51 | 0.05 ± 0.03 | 0.12 ± 0.02 | - | 0.04 ± 0.01 | 0.18 ± 0.19 | - | - | - | 0.06 ± 0.04 | 0.14 ± 0.11 |
| 5-Methyltetradecane | 25117 - 32 - 2 | 1.03 ± 0.19 | 0.60 ± 0.16 | - | - | 0.09 ± 0.02 | - | 0.06 ± 0.02 | - | - | - | - | - | - | - |
| 4,6-Dimethyldodecane | 61141-72-8 | - | 1.38 ± 0.24 | - | 2.51 ± 1.62 | - | 0.08 ± 0.03 | 0.19 ± 0.01 | 0.09 ± 0.04 | 0.22 ± 0.08 | 0.28 ± 0.15 | 0.15 ± 0.05 | 0.10 ± 0.02 | 0.13 ± 0.05 | 0.53 ± 0.48 |
| Heptadecane | 629 - 78 - 7 | 1.99 ± 1.16 | 0.61 ± 0.23 | - | 0.98 ± 0.31 | - | - | 0.09 ± 0.03 | - | 0.05 ± 0.04 | 0.09 ± 0.05 | 0.12 ± 0.03 | 0.16 ± 0.21 | 0.12 ± 0.13 | 0.12 ± 0.07 |
| Pentadecane | 629 - 62 - 9 | 0.65 ± 0.41 | 0.31 ± 0.06 | - | 0.78 ± 0.59 | - | - | 0.05 ± 0.04 | 0.02 ± 0.02 | 0.06 ± 0.06 | - | - | - | - | - |
| 4-Methyltetradecane | 25117 - 24 - 2 | - | - | - | - | - | - | 0.01 ± 0.00 | - | - | - | - | - | 0.03 ± 0.01 | - |
| 3,7-Dimethyldecane | 17312 - 54 - 8 | - | 1.42 ± 0.41 | - | - | - | - | - | - | 0.04 ± 0.03 | - | - | - | - | - |
| 2,5-Dimethylundecane | 17301 - 22 - 3 | - | 0.34 ± 0.21 | - | 0.41 ± 0.10 | - | - | - | - | - | - | - | - | - | - |
| (1-Butylhexyl)benzene | 4537-11-5 | - | 0.10 ± 0.06 | - | - | - | - | - | - | - | - | - | - | - | - |
| Dodecane | 112-40-3 | - | 0.09 ± 0.02 | - | - | - | - | - | - | - | - | - | - | - | - |
| 5-Butylnonane | 17312-63-9 | - | 0.11 ± 0.06 | - | - | - | - | - | - | - | - | - | - | - | - |
| Pseudoionone | 689 - 67 - 8 | - | 0.13 ± 0.05 | - | 0.88 ± 0.35 | 0.06 ± 0.01 | - | 0.02 ± 0.01 | 0.03 ± 0.01 | - | - | - | - | 0.06 ± 0.01 | - |
| 6-Methyl-5-hepten-2-one | 110 - 93 - 0 | 1.04 ± 0.38 | 0.13 ± 0.03 | - | 0.76 ± 0.21 | 0.18 ± 0.02 | 0.18 ± 0.07 | - | 0.11 ± 0.01 | 0.19 ± 0.06 | 0.46 ± 0.14 | - | - | 0.17 ± 0.06 | 0.23 ± 0.07 |
| β-Damascenone | 23726 - 93 - 4 | - | - | - | - | - | - | - | - | - | - | 0.04 ± 0.03 | - | - | - |
| 1-Octen-3-one | 4312-99-6 | - | - | - | - | - | - | - | - | - | - | - | - | 0.15 ± 0.06 | - |
| 2-Methylbutanoic acid | 116-53-0 | - | - | - | - | - | 0.17 ± 0.09 | - | 0.22 ± 0.06 | - | 0.52 ± 0.09 | 0.19 ± 0.14 | - | 0.07 ± 0.03 | - |
| Octanoic acid | 124 - 07 - 2 | - | - | - | 2.38 ± 0.69 | - | - | 0.02 ± 0.00 | - | - | - | - | - | 0.15 ± 0.06 | - |
| Benzoic acid | 65 - 85 - 0 | - | - | - | 0.68 ± 0.28 | - | - | - | 0.05 ± 0.02 | - | - | - | - | - | - |
| Hexanoic acid | 142-62-1 | - | - | - | 6.94 ± 2.19 | - | - | - | - | - | - | - | - | 0.13 ± 0.02 | - |
| Nonanoic acid | 112 - 05 - 0 | - | - | - | 10.01 ± 5.52 | - | - | - | - | - | - | - | - | - | - |
| 2,4-Di-tert-butylphenol | 96-76-4 | 4.22 ± 1.24 | 0.89 ± 0.45 | 0.75 ± 0.32 | 2.35 ± 0.83 | - | 0.23 ± 0.04 | 0.14 ± 0.06 | 0.26 ± 0.05 | 0.58 ± 0.64 | 0.83 ± 0.58 | 0.66 ± 0.12 | 0.53 ± 0.07 | 0.30 ± 0.07 | 0.42 ± 0.12 |
| N,N-Dibutylformamide | 761 - 65 - 9 | - | - | - | - | - | - | - | - | - | 0.18 ± 0.14 | - | 0.07 ± 0.03 | 0.02 ± 0.01 | - |
| α-Terpineol | 98-55-5 | 1.73 ± 1.46 | 0.33 ± 0.03 | - | - | - | 0.10 ± 0.01 | 0.03 ± 0.01 | 0.08 ± 0.02 | 0.12 ± 0.06 | 0.36 ± 0.09 | 0.16 ± 0.01 | - | 0.07 ± 0.03 | 0.07 ± 0.01 |
| Linalool | 78-70-6 | 9.95 ± 15.69 | 2.48 ± 0.68 | - | 3.94 ± 2.17 |  | 0.11 ± 0.01 | 0.03 ± 0.01 | 0.10 ± 0.03 | 0.16 ± 0.07 | 0.27 ± 0.07 | 0.16 ± 0.06 | 0.10 ± 0.03 | 0.12 ± 0.03 | 0.15 ± 0.05 |
| trans-Ocimenol | 7643-60-9 | 4.61 ± 1.29 | 0.20 ± 0.04 | 0.61 ± 0.11 | - | - | 0.19 ± 0.02 | 0.06 ± 0.04 | - | - | - | 0.37 ± 0.06 | 0.76 ± 0.04 | - | 0.08 ± 0.05 |
| (Z)-Linalool oxide (furanoid) | 5989 - 33 - 3 | 1.46 ± 1.52 | - | - | - | - | - | - | - | 0.34 ± 0.12 | - | 0.10 ± 0.04 | 0.22 ± 0.13 | 0.03 ± 0.02 | 0.09 ± 0.03 |
| Geranic oxide | 7392 - 19 - 0 | 3.82 ± 0.48 | - | 0.56 ± 0.08 | - | - | - | 0.11 ± 0.02 | - | - | - | - | 0.61 ± 0.09 | - | - |
| trans-Linalool oxide (furanoid) | 34995 - 77 - 2 | - | - | - | - | - | - | - | 0.16 ± 0.17 | - | 1.23 ± 0.96 | - | - | 0.04 ± 0.02 | - |
| Ocimene quintoxide | 7416 - 35 - 5 | 1.55 ± 0.30 | 0.13 ± 0.01 | 0.29 ± 0.05 | - | - | - | - | - | - | - | - | 0.11 ± 0.05 | - | - |
| α-Pinene | 80 - 56 - 8 | - | - | - | 1.50 ± 0.22 | - | - | - | - | - | - | - | - | - | - |
| β-Myrcene | 123-35-3 | - | 0.20 ± 0.07 | - | - | - | - | - | - | - | - | - | - | - | - |

Notes: VOCs, volatile organic compounds. Values are presented as mean ± standard deviation (SD) of three independent biological replicates (n = 3) and expressed as μg/g. “−” = not detected.

**Table S4.** Detailed assignment of volatile organic compounds (VOCs) to weighted correlation network analysis (WCNA) modules.

| **Compound** | **Chemical class** | **WCNA module** |
| --- | --- | --- |
| Butanoic acid, 2-methyl- | Acids | MEblack |
| 1-Hexanol, 2-ethyl- | Alcohols | MEblack |
| 1-Nonanol | Alcohols | MEblack |
| Phenylethyl Alcohol | Alcohols | MEblack |
| 1-Butanol, 3-methyl- | Alcohols | MEblack |
| 7-Octen-2-ol, 2-methyl-6-methylene- | Alcohols | MEblue |
| Benzaldehyde, 2,4-dimethyl- | Aldehydes | MEblue |
| Nonanal | Aldehydes | MEblue |
| Hexadecane | Alkanes | MEblue |
| Tetradecane | Alkanes | MEblue |
| Tetradecane, 5-methyl- | Alkanes | MEblue |
| Heptadecane | Alkanes | MEblue |
| Pentadecane | Alkanes | MEblue |
| 2H-Pyran, 2-ethenyltetrahydro-2,6,6-trimethyl- | Terpenoids | MEblue |
| 2-Furanmethanol, 5-ethenyltetrahydro-.alpha.,.alpha.,5-trimethyl-, cis- | Terpenoids | MEblue |
| Furan, tetrahydro-2,2-dimethyl-5-(1-methyl-1-propenyl)- | Terpenoids | MEblue |
| 5-Hepten-2-one, 6-methyl- | Ketones | MEblue |
| 2,4-Di-tert-butylphenol | Others | MEblue |
| .alpha.-Terpineol | Terpenoids | MEblue |
| Linalool | Terpenoids | MEblue |
| trans-Ocimenol | Terpenoids | MEblue |
| 1-Octanol | Alcohols | MEbrown |
| 1-Heptanol | Alcohols | MEbrown |
| 1-Hexanol | Alcohols | MEbrown |
| 1-Propanol, 2-methyl- | Alcohols | MEbrown |
| Benzoic acid, methyl ester | Esters | MEbrown |
| Decanoic acid, ethyl ester | Esters | MEbrown |
| Dodecanoic acid, ethyl ester | Esters | MEbrown |
| Hexanoic acid, ethyl ester | Esters | MEbrown |
| Furan, 2-butyltetrahydro- | Furans | MEbrown |
| 2-Buten-1-one, 1-(2,6,6-trimethyl-1,3-cyclohexadien-1-yl)-, (E)- | Terpenoids | MEbrown |
| 2-Nonenal, (E)- | Aldehydes | MEgreen |
| 2-Octenal, (E)- | Aldehydes | MEgreen |
| Citral | Aldehydes | MEgreen |
| Hexanal | Aldehydes | MEgreen |
| Tetradecane, 4-methyl- | Alkanes | MEgreen |
| 1-Octen-3-one | Ketones | MEgreen |
| 1-Octen-3-ol | Alcohols | MEgrey |
| Acetic acid, nonyl ester | Esters | MEgrey |
| Ethyl Acetate | Esters | MEgrey |
| Acetic acid, hexyl ester | Esters | MEgrey |
| Furan, tetrahydro-2,2,5,5-tetramethyl- | Furans | MEgrey |
| 2(3H)-Furanone, 5-hexyldihydro- | Furans | MEgrey |
| Decane, 3,7-dimethyl- | Alkanes | MEpink |
| Dodecane | Alkanes | MEpink |
| Nonane, 5-butyl- | Alkanes | MEpink |
| Benzene, (1-butylhexyl)- | Others | MEpink |
| .beta.-Myrcene | Terpenoids | MEpink |
| 2-Octen-1-ol, (Z)- | Alcohols | MEred |
| 1,5,7-Octatrien-3-ol, 3,7-dimethyl- | Alcohols | MEred |
| Formamide, N,N-dibutyl- | Others | MEred |
| Acetic acid, 2-phenylethyl ester | Esters | MEred |
| trans-Linalool oxide (furanoid) | Terpenoids | MEred |
| Octanoic acid | Acids | MEturquoise |
| Benzoic acid | Acids | MEturquoise |
| Hexanoic acid | Acids | MEturquoise |
| Nonanoic acid | Acids | MEturquoise |
| 2,4-Heptadienal, (E,E)- | Aldehydes | MEturquoise |
| 2-Heptenal, (E)- | Aldehydes | MEturquoise |
| Octanal | Aldehydes | MEturquoise |
| Dodecane, 4-methyl- | Alkanes | MEturquoise |
| Dodecane, 4,6-dimethyl- | Alkanes | MEturquoise |
| Undecane, 2,5-dimethyl- | Alkanes | MEturquoise |
| Octanoic acid, ethyl ester | Esters | MEturquoise |
| Propanoic acid, 2-methyl-, 3-hydroxy-2,2,4-trimethylpentyl ester | Esters | MEturquoise |
| Hexanoic acid, butyl ester | Esters | MEturquoise |
| Hexanoic acid, hexyl ester | Esters | MEturquoise |
| 5,9-Undecadien-2-one, 6,10-dimethyl- | Terpenoids | MEturquoise |
| .alpha.-Pinene | Terpenoids | MEturquoise |
| Benzyl alcohol | Alcohols | MEyellow |
| Benzaldehyde | Aldehydes | MEyellow |
| Benzoic acid, ethyl ester | Esters | MEyellow |
| Acetic acid, phenylmethyl ester | Esters | MEyellow |
| Hexadecanoic acid, ethyl ester | Esters | MEyellow |
| Methyl salicylate | Esters | MEyellow |

Note: A total of 75 VOCs were assigned to eight co-abundance modules (MEblack, MEblue, MEbrown, MEgreen, MEyellow, MEpink, MEred, and MEturquoise). MEgrey represents VOCs that were not assigned to a specific co-abundance module. VOCs, volatile organic compounds; WCNA, weighted correlation network analysis.

**Supplementary Table S5.** OAV values of identified volatile organic compounds (VOCs) in candied *Prunus mume* (CPM) from 14 origins.

| **Name** | **CAS** | **Odor Threshold** | **MB** | | **PW** | | **DY** | | **DZ** | **CZ** | | **JY** | | **SG** | | **CX** | | **DL** | | **LJ** | | **ZZ** | | **FZ** | | **SX** | | **HZ** | |  |
| --- | --- | --- | --- | --- | --- | --- | --- | --- | --- | --- | --- | --- | --- | --- | --- | --- | --- | --- | --- | --- | --- | --- | --- | --- | --- | --- | --- | --- | --- | --- |
| 2-Ethyl-1-hexanol | 104 - 76 - 7 | 0.27 | - | | - | | - | | - | 0.15 | | 0.26 | | 0.07 | | 0.26 | | 0.22 | | 0.41 | | 0.33 | | 0.41 | | 0.19 | | 0.22 | |  |
| 1-Nonanol | 143 - 08 - 8 | 0.08 | - | | - | | - | | - | 1.13 | | - | | 0.5 | | - | | - | | 2 | | 1.75 | | 0.75 | | - | | - | |  |
| 1-Octanol | 111 - 87 - 5 | 0.1 | - | | - | | - | | - | 1.2 | | 3.6 | | 0.5 | | 0.9 | | 0.9 | | - | | 2.5 | | 1.4 | | 0.6 | | 1.6 | |  |
| 1-Octen-3-ol | 3391 - 86 - 4 | 0.0015 | - | | - | | - | | - | 133.33 | | 100 | | - | | 66.67 | | - | | - | | - | | - | | - | | - | |  |
| (Z)-2-Octen-1-ol | 26001 - 58 - 1 | - | - | | - | | - | | - | - | | - | | - | | - | | - | | - | | - | | - | | - | | - | |  |
| Benzyl alcohol | 100 - 51 - 6 | 0.62 | - | | - | | 0.37 | | 0.56 | 1.61 | | 0.44 | | 0.08 | | 11.58 | | 16.08 | | 2.74 | | 0.55 | | 0.58 | | 1.52 | | 7.69 | |  |
| Phenylethyl alcohol | 60-12-8 | 0.086 | - | | - | | - | | - | 7.21 | | 32.91 | | 3.26 | | 7.79 | | 14.53 | | 41.16 | | 31.63 | | 5.81 | | 7.56 | | 3.6 | |  |
| α-Terpineol | 98-55-5 | 0.0046 | 376.09 | | 71.74 | | - | | - | - | | 21.74 | | 6.52 | | 17.39 | | 26.09 | | 78.26 | | 34.78 | | - | | 15.22 | | 15.22 | |  |
| 3-Methyl-1-butanol | 123-51-3 | 0.071 | - | | - | | - | | - | - | | 83.52 | | 11.27 | | 36.2 | | 45.49 | | 107.18 | | 91.97 | | 14.93 | | 27.89 | | 10.14 | |  |
| 1-Heptanol | 111-70-6 | 0.0054 | - | | - | | - | | - | - | | 35.19 | | 7.41 | | - | | - | | - | | - | | - | | - | | 27.78 | |  |
| 1-Hexanol | 111-27-3 | 0.15 | - | | - | | - | | - | - | | 5.33 | | 0.33 | | - | | - | | - | | 5.4 | | 6.33 | | 1.47 | | - | |  |
| 2-Methyl-1-propanol | 78-83-1 | 1 | - | | - | | - | | - | - | | 1.59 | | - | | - | | - | | - | | 1.46 | | - | | - | | - | |  |
| 2,4-Di-tert-butylphenol | 96-76-4 | 0.5 | 8.44 | | 1.78 | | 1.5 | | 4.7 | - | | 0.46 | | 0.28 | | 0.52 | | 1.16 | | 1.66 | | 1.32 | | 1.06 | | 0.6 | | 0.84 | |  |
| 7-Octen-2-ol, 2-methyl-6-methylene- | 543-39-5 | - | - | | - | | - | | - | - | | - | | - | | - | | - | | - | | - | | - | | - | | - | |  |
| Linalool | 78-70-6 | 0.0015 | 6633.33 | | 1653.33 | | - | | 2626.67 | - | | 73.33 | | 20 | | 66.67 | | 106.67 | | 180 | | 106.67 | | 66.67 | | 80 | | 100 | |  |
| trans-Ocimenol | 7643-60-9 | - | - | | - | | - | | - | - | | - | | - | | - | | - | | - | | - | | - | | - | | - | |  |
| 1,5,7-Octatrien-3-ol, 3,7-dimethyl- | 29957 - 43 - 5 | - | - | | - | | - | | - | - | | - | | - | | - | | - | | - | | - | | - | | - | | - | |  |
| (Z)-Linalool oxide (furanoid) | 5989 - 33 - 3 | 0.1 | 14.6 | | - | | - | | - | - | | - | | - | | - | | 3.4 | | - | | 1 | | 2.2 | | 0.3 | | 0.9 | |  |
| (E,E)-2,4-Heptadienal | 4313-03-5 | 0.02955 | - | | - | | - | | 18.27 | 6.43 | | 8.12 | | - | | 3.72 | | 8.8 | | 13.2 | | - | | - | | 5.75 | | 6.09 | |  |
| 6,10-Dimethyl-5,9-undecadien-2-one | 689 - 67 - 8 | 0.01 | - | | 13 | | - | | 88 | 6 | | - | | 2 | | 3 | | - | | - | | - | | - | | 6 | | - | |  |
| 6-Methyl-5-hepten-2-one | 110 - 93 - 0 | 0.05 | 20.8 | | 2.6 | | - | | 15.2 | 3.6 | | 3.6 | | - | | 2.2 | | 3.8 | | 9.2 | | - | | - | | 3.4 | | 4.6 | |  |
| Benzaldehyde | 100 - 52 - 7 | 0.35 | - | | - | | 7.17 | | - | 5.43 | | - | | - | | 30.89 | | 41.74 | | - | | - | | - | | 0.34 | | 14.46 | |  |
| 2,4-Dimethylbenzaldehyde | 15764 - 16 - 6 | - | - | | - | | - | | - | - | | - | | - | | - | | - | | - | | - | | - | | - | | - | |  |
| 2,2,5,5-Tetramethyltetrahydrofuran | 15045 - 43 - 9 | - | - | | - | | - | | - | - | | - | | - | | - | | - | | - | | - | | - | | - | | - | |  |
| Nonanal | 124 - 19 - 6 | 0.0028 | 342.86 | | 53.57 | | - | | 221.43 | 35.71 | | 46.43 | | - | | - | | - | | - | | - | | - | | - | | - | |  |
| 2(3H)-Furanone, 5-hexyldihydro- | 706-14-9 | 0.0011 | - | | 100 | | - | | - | - | | 163.64 | | 27.27 | | - | | - | | 136.36 | | - | | 400 | | 63.64 | | - | |  |
| (E)-2-Heptenal | 18829-55-5 | 0.013 | - | | - | | - | | 77.69 | - | | 12.31 | | 3.08 | | 10.77 | | 10.77 | | 17.69 | | - | | - | | 18.46 | | 21.54 | |  |
| 2-Butyltetrahydrofuran | 1004-29-1 | - | - | | - | | - | | - | - | | - | | - | | - | | - | | - | | - | | - | | - | | - | |  |
| Geranic oxide | 7392 - 19 - 0 | - | - | | - | | - | | - | - | | - | | - | | - | | - | | - | | - | | - | | - | | - | |  |
| trans-Linalool oxide (furanoid) | 34995 - 77 - 2 | 0.19 | - | | - | | - | | - | - | | - | | - | | 0.84 | | - | | 6.47 | | - | | - | | 0.21 | | - | |  |
| N,N-Dibutylformamide | 761 - 65 - 9 | - | - | | - | | - | | - | - | | - | | - | | - | | - | | - | | - | | - | | - | | - | |  |
| β-Damascenone | 23726 - 93 - 4 | 2e-06 | - | | - | | - | | - | - | | - | | - | | - | | - | | - | | 20000 | | - | | - | | - | |  |
| Ocimene quintoxide | 7416 - 35 - 5 | - | - | | - | | - | | - | - | | - | | - | | - | | - | | - | | - | | - | | - | | - | |  |
| 1-Octen-3-one | 4312-99-6 | 5e-06 | - | | - | | - | | - | - | | - | | - | | - | | - | | - | | - | | - | | 30000 | | - | |  |
| (E)-2-Nonenal | 18829-56-6 | 9e-05 | - | | - | | - | | - | - | | - | | - | | - | | - | | - | | - | | - | | 444.44 | | - | |  |
| (E)-2-Octenal | 2548-87-0 | 0.003 | - | | - | | - | | - | - | | - | | - | | - | | - | | - | | - | | - | | 93.33 | | - | |  |
| Citral | 5392-40-5 | 0.028 | - | | - | | - | | - | - | | - | | - | | - | | - | | - | | - | | - | | 1.43 | | - | |  |
| Hexanal | 66-25-1 | 0.005 | - | | - | | - | | - | - | | - | | - | | - | | - | | - | | - | | - | | 34 | | - | |  |
| Octanal | 124-13-0 | 0.00088 | - | | - | | - | | 375 | - | | - | | - | | - | | - | | - | | - | | - | | 56.82 | | - | |  |
| Methyl benzoate | 93 - 58 - 3 | 0.00052 | - | | - | | - | | - | 76.92 | | 153.85 | | 38.46 | | - | | - | | - | | 173.08 | | 211.54 | | - | | 57.69 | |  |
| Ethyl benzoate | 93-89-0 | 0.053 | - | | - | | - | | - | - | | 3.21 | | - | | 5.09 | | 5.28 | | - | | - | | - | | 2.08 | | 1.32 | |  |
| Ethyl decanoate | 110-38-3 | 0.005 | - | | - | | - | | - | - | | 14 | | 8 | | 10 | | 32 | | - | | 38 | | - | | 8 | | - | |  |
| Ethyl dodecanoate | 106-33-2 | 0.4 | - | | - | | - | | - | - | | 0.18 | | 0.05 | | 0.2 | | 0.18 | | - | | 0.3 | | - | | - | | - | |  |
| Nonyl acetate | 143 - 13 - 5 | - | - | | - | | - | | - | - | | - | | - | | - | | - | | - | | - | | - | | - | | - | |  |
| Ethyl acetate | 141 - 78 - 6 | 0.005 | - | | - | | - | | - | - | | - | | 222 | | - | | - | | - | | - | | 144 | | - | | - | |  |
| Ethyl octanoate | 106 - 32 - 1 | 0.015 | - | | - | | - | | 28.67 | - | | - | | 2.67 | | - | | - | | - | | 12.67 | | - | | 6 | | - | |  |
| 3-Hydroxy-2,2,4-trimethylpentyl isobutyrate | 77 - 68 - 9 | - | - | | - | | - | | - | - | | - | | - | | - | | - | | - | | - | | - | | - | | - | |  |
| 2-Phenylethyl acetate | 103-45-7 | 0.019 | - | | - | | - | | - | - | | - | | - | | 2.11 | | 4.21 | | 16.84 | | - | | - | | - | | - | |  |
| Benzyl acetate | 140-11-4 | 0.03 | - | | - | | - | | - | - | | - | | - | | 3.33 | | 7 | | - | | - | | - | | - | | 5.33 | |  |
| Ethyl hexadecanoate | 628 - 97 - 7 | 2 | - | | - | | - | | - | - | | - | | - | | 0.01 | | - | | - | | - | | - | | - | | - | |  |
| Methyl salicylate | 119 - 36 - 8 | 0.04 | - | | - | | - | | - | - | | - | | - | | 1.75 | | - | | - | | - | | - | | - | | - | |  |
| Ethyl hexanoate | 123-66-0 | 0.01 | - | | - | | - | | - | - | | - | | - | | - | | - | | - | | 29 | | - | | - | | - | |  |
| Hexyl acetate | 142 - 92 - 7 | 1 | - | | - | | - | | - | - | | - | | - | | - | | - | | - | | - | | 0.36 | | - | | - | |  |
| Butyl hexanoate | 626 - 82 - 4 | 0.7 | - | | - | | - | | 2.86 | - | | - | | - | | - | | - | | - | | - | | - | | - | | - | |  |
| Hexyl hexanoate | 6378 - 65 - 0 | 6.4 | - | | - | | - | | 0.07 | - | | - | | - | | - | | - | | - | | - | | - | | - | | - | |  |
| 4-Methyldodecane | 6117 - 97 - 1 | - | - | | - | | - | | - | - | | - | | - | | - | | - | | - | | - | | - | | - | | - | |  |
| Hexadecane | 544 - 76 - 3 | - | - | | - | | - | | - | - | | - | | - | | - | | - | | - | | - | | - | | - | | - | |  |
| Tetradecane | 629 - 59 - 4 | 1 | 1.59 | | - | | - | | 0.82 | 0.05 | | 0.12 | | - | | 0.04 | | 0.18 | | - | | - | | - | | 0.06 | | 0.14 | |  |
| 5-Methyltetradecane | 25117 - 32 - 2 | - | - | | - | | - | | - | - | | - | | - | | - | | - | | - | | - | | - | | - | | - | |  |
| 4,6-Dimethyldodecane | 61141-72-8 | - | - | - | | - | | - | | | - | | - | | - | | - | | - | | - | | - | | - | | - | | - | |
| Heptadecane | 629 - 78 - 7 | - | - | - | | - | | - | | | - | | - | | - | | - | | - | | - | | - | | - | | - | | - | |
| Pentadecane | 629 - 62 - 9 | - | - | - | | - | | - | | | - | | - | | - | | - | | - | | - | | - | | - | | - | | - | |
| 4-Methyltetradecane | 25117 - 24 - 2 | - | - | - | | - | | - | | | - | | - | | - | | - | | - | | - | | - | | - | | - | | - | |
| 3,7-Dimethyldecane | 17312 - 54 - 8 | - | - | - | | - | | - | | | - | | - | | - | | - | | - | | - | | - | | - | | - | | - | |
| α-Pinene | 80 - 56 - 8 | 0.006 | - | - | | - | | 250 | | | - | | - | | - | | - | | - | | - | | - | | - | | - | | - | |
| 2,5-Dimethylundecane | 17301 - 22 - 3 | - | - | - | | - | | - | | | - | | - | | - | | - | | - | | - | | - | | - | | - | | - | |
| β-Myrcene | 123-35-3 | 0.015 | - | 13.33 | | - | | - | | | - | | - | | - | | - | | - | | - | | - | | - | | - | | - | |
| (1-Butylhexyl)benzene | 4537-11-5 | - | - | - | | - | | - | | | - | | - | | - | | - | | - | | - | | - | | - | | - | | - | |
| Dodecane | 112-40-3 | 10 | - | | 0.01 | | - | | - | - | | - | | - | | - | | - | | - | | - | | - | | - | | - | |  |
| 5-Butylnonane | 17312-63-9 | - | - | | - | | - | | - | - | | - | | - | | - | | - | | - | | - | | - | | - | | - | |  |
| 2-Methylbutanoic acid | 116-53-0 | 0.1 | - | | - | | - | | - | - | | 1.7 | | - | | 2.2 | | - | | 5.2 | | 1.9 | | - | | 0.7 | | - | |  |
| Octanoic acid | 124 - 07 - 2 | 1.405 | - | | - | | - | | 1.69 | - | | - | | 0.01 | | - | | - | | - | | - | | - | | 0.11 | | - | |  |
| Benzoic acid | 65 - 85 - 0 | - | - | | - | | - | | - | - | | - | | - | | - | | - | | - | | - | | - | | - | | - | |  |
| Hexanoic acid | 142-62-1 | 0.89 | - | | - | | - | | 7.8 | - | | - | | - | | - | | - | | - | | - | | - | | 0.15 | | - | |  |

Notes: VOCs, volatile organic compounds; OAV, odor activity value. Odor threshold values were obtained from Compilations of Odor Threshold Values in Air, Water and Other Media (Edition 2011; Boelens Aroma Chemical Information Service). The OAV was calculated as the ratio of the concentration of each volatile compound in CPM to its corresponding odor threshold in water. Compounds with OAV ≥ 1 were considered aroma-active and likely to contribute to the overall aroma profile. Values were calculated from three independent biological replicates (n = 3). “−” = not detected.
